# Loss of CSL Unlocks a Hypoxic Response and Enhanced Tumor Growth Potential in Breast Cancer Cells

**DOI:** 10.1016/j.stemcr.2016.03.004

**Published:** 2016-04-07

**Authors:** Eike-Benjamin Braune, Yat Long Tsoi, Yee Peng Phoon, Sebastian Landor, Helena Silva Cascales, Daniel Ramsköld, Qiaolin Deng, Arne Lindqvist, Xiaojun Lian, Cecilia Sahlgren, Shao-Bo Jin, Urban Lendahl

**Affiliations:** 1 Department of Cell and Molecular Biology, Karolinska Institutet, 17177 Stockholm, Sweden; 2 Turku Centre for Biotechnology, Abo Akademi University and University of Turku, 20520 Turku, Finland; 3 Rheumatology Unit, Department of Medicine Solna, Karolinska Institutet, Karolinska University Hospital, 17176 Stockholm, Sweden

**Keywords:** breast cancer, Notch signaling, xenograft, transcriptome, mitosis

## Abstract

Notch signaling is an important regulator of stem cell differentiation. All canonical Notch signaling is transmitted through the DNA-binding protein CSL, and hyperactivated Notch signaling is associated with tumor development; thus it may be anticipated that CSL deficiency should reduce tumor growth. In contrast, we report that genetic removal of CSL in breast tumor cells caused accelerated growth of xenografted tumors. Loss of CSL unleashed a hypoxic response during normoxic conditions, manifested by stabilization of the HIF1α protein and acquisition of a polyploid giant-cell, cancer stem cell-like, phenotype. At the transcriptome level, loss of CSL upregulated more than 1,750 genes and less than 3% of those genes were part of the Notch transcriptional signature. Collectively, this suggests that CSL exerts functions beyond serving as the central node in the Notch signaling cascade and reveals a role for CSL in tumorigenesis and regulation of the cellular hypoxic response.

## Highlights

• Loss of CSL accelerates tumor growth
• CSL deficiency unleashes a hypoxic response during normoxia
• Loss of CSL leads to a polyploid giant-cell, cancer stem cell-like morphology
• CSL-deficient cells show a Notch-independent transcriptional signature

## Teaser

In this report, Lendahl, Jin, and colleagues show that the loss of CSL (RBP-Jk) promotes tumor growth in vivo and provide evidence for a role of CSL in controlling the cellular hypoxic response and cell cycle/cytokinesis. Furthermore, they show that loss of CSL acts beyond only mediating Notch signaling.

## Introduction

In most cellular contexts Notch signaling acts as a gatekeeper to differentiation, promoting maintenance of stem or progenitor cell fates (Andersson et al., 2011, Guruharsha et al., 2012). Modulation of Notch signaling is used to control stem or progenitor cell differentiation in vitro, for example toward neural, intestinal, or hematopoietic lineages (Lowell et al., 2006, Schmitt et al., 2004, Yin et al., 2014). Deregulated Notch signaling is increasingly linked to cancer, and Notch receptor mutations are found in, for example, T cell leukemia, non-small cell lung cancer, and breast cancer as well as in several types of tumor cell lines (Mutvei et al., 2015, Robinson et al., 2011, Weng et al., 2004, Westhoff et al., 2009). Notch signaling is also frequently hyperactivated in a range of tumors, including breast cancer (for review see Andersson and Lendahl, 2014).

Notch signaling ensues when transmembrane Notch ligands of the Jagged or Delta-like type interact with Notch receptors on a juxtaposed cell. This results in proteolytic cleavage and liberation of the intracellular domain of the Notch receptor (Notch ICD), which relocates to the cell nucleus and interacts with the DNA-binding protein CSL (also known as RBP-Jk or CBF1), thus making CSL the central node in the signaling cascade for all four Notch receptors (Notch 1–4) (Andersson et al., 2011). In the “Notch off” state, CSL acts as a repressor and binds a number of transcriptional co-repressors, such as SHARP/MINT, KDM5A, and KyoT2 (for review see Borggrefe and Oswald, 2014). In the “Notch on” state, i.e., upon binding to Notch ICD, CSL sheds the co-repressors and instead recruits co-activators, such as p300 and PCAF, converting it to an activator. The interaction between Notch ICD and CSL is stabilized by the MAML protein, and the ternary Notch ICD/MAML/CSL complex induces expression of Notch downstream genes (Nam et al., 2006, Wilson and Kovall, 2006). It has traditionally been assumed that CSL serves as a DNA-bound repressor in the absence of Notch, and in line with this, CSL can bind to DNA in the absence of Notch and remains bound to DNA even during mitosis (Lake et al., 2014). Recent studies, however, provide support for a more dynamic view whereby CSL is recruited to the DNA by Notch ICD (Castel et al., 2013, Krejcí and Bray, 2007).

It is an open question whether CSL only transmits the signal from the Notch receptors or also plays a role in other, non-Notch-related signaling transductions. Gene-targeting experiments show that phenotypes resulting from targeting of Notch ligands or receptors in some situations are phenocopied by targeting of CSL, for example during somitogenesis (Conlon et al., 1995, Oka et al., 1995) or in memory T cells (Maekawa et al., 2015), which is in line with CSL functioning exclusively as the central hub in the Notch signaling cascade (Guruharsha et al., 2012). On the other hand, there are also an increasing number of proteins, such as CTCF, EBNA3c, interferon regulatory factor 4, and RITA (see Collins et al., 2014 and references therein), which are not part of the Notch signaling mechanism but interact with CSL, suggesting that CSL has a broader range of actions extending beyond only transmitting Notch signaling.

In this study, we address the question of possible additional roles for CSL and report the unexpected discovery that transplanted breast tumor cells in which CSL was genetically ablated caused rapid tumor growth, a phenotype opposite to blocking Notch function at the receptor level. The phenotype was accompanied by acquisition of a hypoxic response during normoxia and a polyploid giant-cell, cancer stem cell-like, morphology.

## Results

### Loss of CSL Promotes Tumor Growth In Vivo

**Figure 1:**
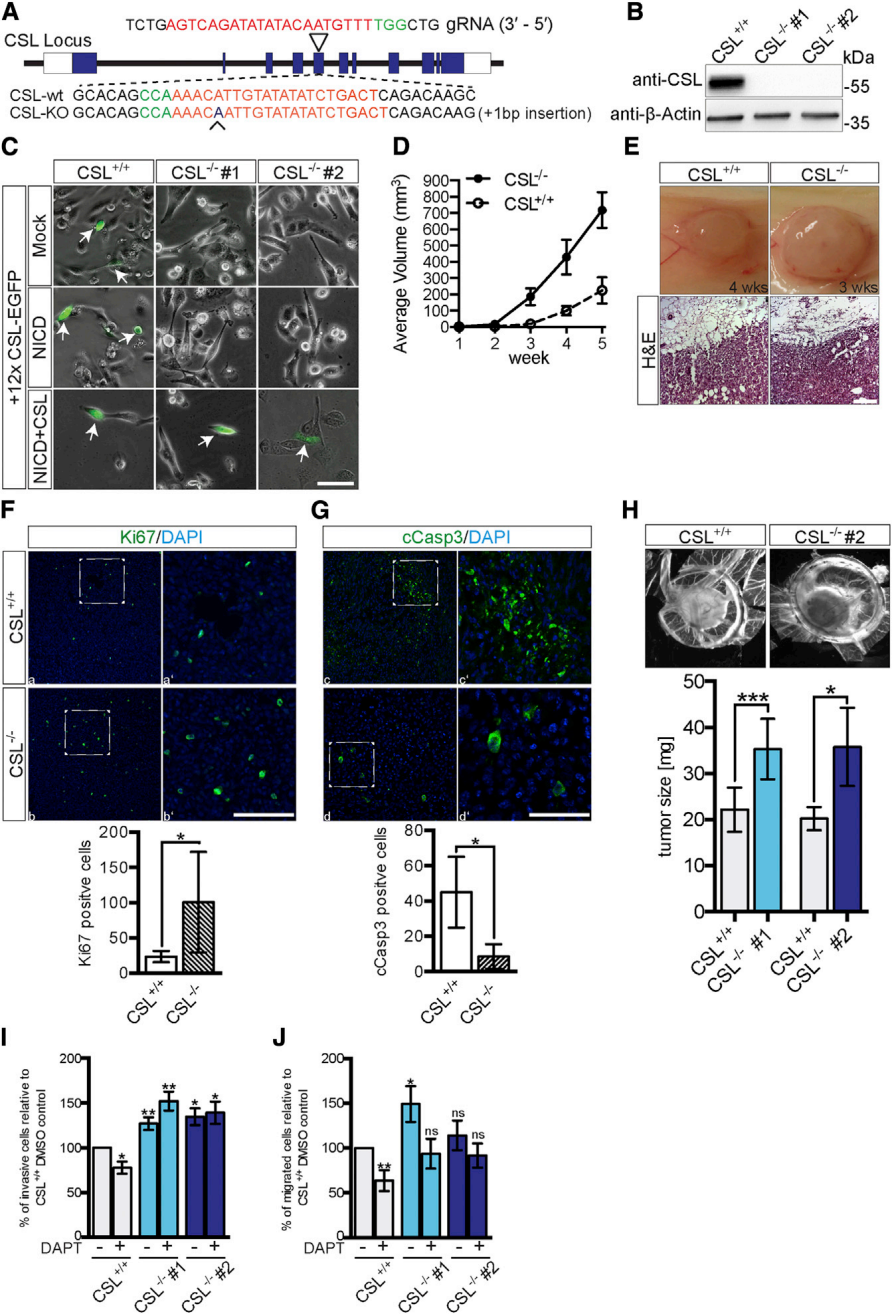
CSL-Deficient Cells Accelerate Tumor Growth In Vivo (A) Schematic representation of CRISPR/Cas9 targeting of the CSL locus. The triangle points to the targeted exon. Red letters represent the guide RNA sequence and green letters the PAM sequence. (B) Western blot of CSL and β-actin (loading control) in control (CSL^+/+^) and two clones of CSL-deficient MDA-MB-231 cells (CSL^−/−^). (C) Notch reporter (12x CSL-EGFP) activity in control and CSL-deficient cells after transfection of 12xCSL-EGFP, Notch1-ICD (NICD), and CSL, as indicated. White arrows indicate cells expressing EGFP. (D) Average tumor volume at different time points after xenografting CSL^+/+^ or CSL^−/−^ cells. Eight tumors of four mice per group were analyzed. (E) Representative images and H&E stainings of control and CSL-deficient tumors. (F and G) Analysis of Ki67 (F) and cleaved Caspase-3 (cCasp3) (G) expression in MDA-MB-231^CSL+/+^ and CSL-deficient tumor sections (enlarged images to the right). At the bottom of each figure, the number of positive cells is quantified. Signals of at least four randomly chosen images from one tumor sample of each kind were counted. (H) Analysis and quantification of tumor growth in the chick chorioallantoic membrane (CAM) assay for CSL^+/+^ and CSL^−/−^ cells. At least five different tumors of each kind were measured. (I and J) Invasion and migration assays for CSL^+/+^ and CSL^−/−^ cells. This analysis is based on at least three independent experiments. Data are shown as percent of wild-type MDA-MB-231 DMSO control cells (set to 100%). Data are presented as mean ± SEM. ^∗^p ≤ 0.05; ^∗∗^p ≤ 0.01; ^∗∗∗^p ≤ 0.001. ns, not significant. Scale bars: 100 μm (C), 200 μm (E lower), 100 μm (F and G), and 75 μm (F and G inset).

To explore the role of CSL in a breast tumor context, we targeted both CSL alleles by CRISPR/Cas9 genome editing in MDA-MB-231 cells (Figure 1A), a breast tumor cell line with active Notch signaling and which promotes tumor growth when transplanted in vivo (Holliday and Speirs, 2011, Jin et al., 2013). In the two independent MDA-MB-231^CSL−/−^ clones selected for further analysis, there was as expected no detectable CSL protein (Figure 1B), and the activity of a Notch reporter construct (12x CSL-EGFP) (Hansson et al., 2006) was abrogated (Figure 1C). Reintroduction of CSL into the MDA-MB-231^CSL−/−^ cells restored Notch reporter activity (Figure 1C) as well as expression of established Notch downstream genes (Figure S1).

Transplantation of the MDA-MB-231^CSL−/−^ cells into the mammary fat pad in mice resulted in accelerated tumor growth compared with control MDA-MB-231^CSL+/+^ cells. The difference was already noticeable after 3 weeks, and after 5 weeks the tumor volume from the MDA-MB-231^CSL−/−^ cells was 2.8 times larger than in the control cell line (Figures 1D and 1E). Proliferation was increased and apoptosis decreased in the MDA-MB-231^CSL−/−^ tumors as determined by Ki67 (Figure 1F) and cleaved Caspase-3 (Figure 1G) staining, respectively. To assess tumor growth potential in an alternative manner, we cultured both CSL-deficient clones on the chorioallantoic membrane in eggs, and tumor growth was robustly enhanced for both clones (Figure 1H).

In keeping with the tumor data, both CSL^−/−^ clones displayed elevated penetration in a Matrigel invasion assay (Figure 1I). In a transwell migration assay, clone #1 showed enhanced migration whereas migration was not significantly changed in clone #2 (Figure 1J). Treatment with the γ-secretase inhibitor DAPT, which blocks receptor cleavage and thus Notch1 ICD generation, inhibited cell migration and reduced the invasion of CSL^+/+^ but not of CSL-deficient cells (Figures 1I and 1J). In conclusion, these data show that removal of CSL enhances tumor growth in vivo and invasiveness in vitro, and exerts an effect distinct from blockade at the Notch receptor level.

### Loss of CSL Unleashes a Hypoxic Response under Normoxic Conditions

**Figure 2:**
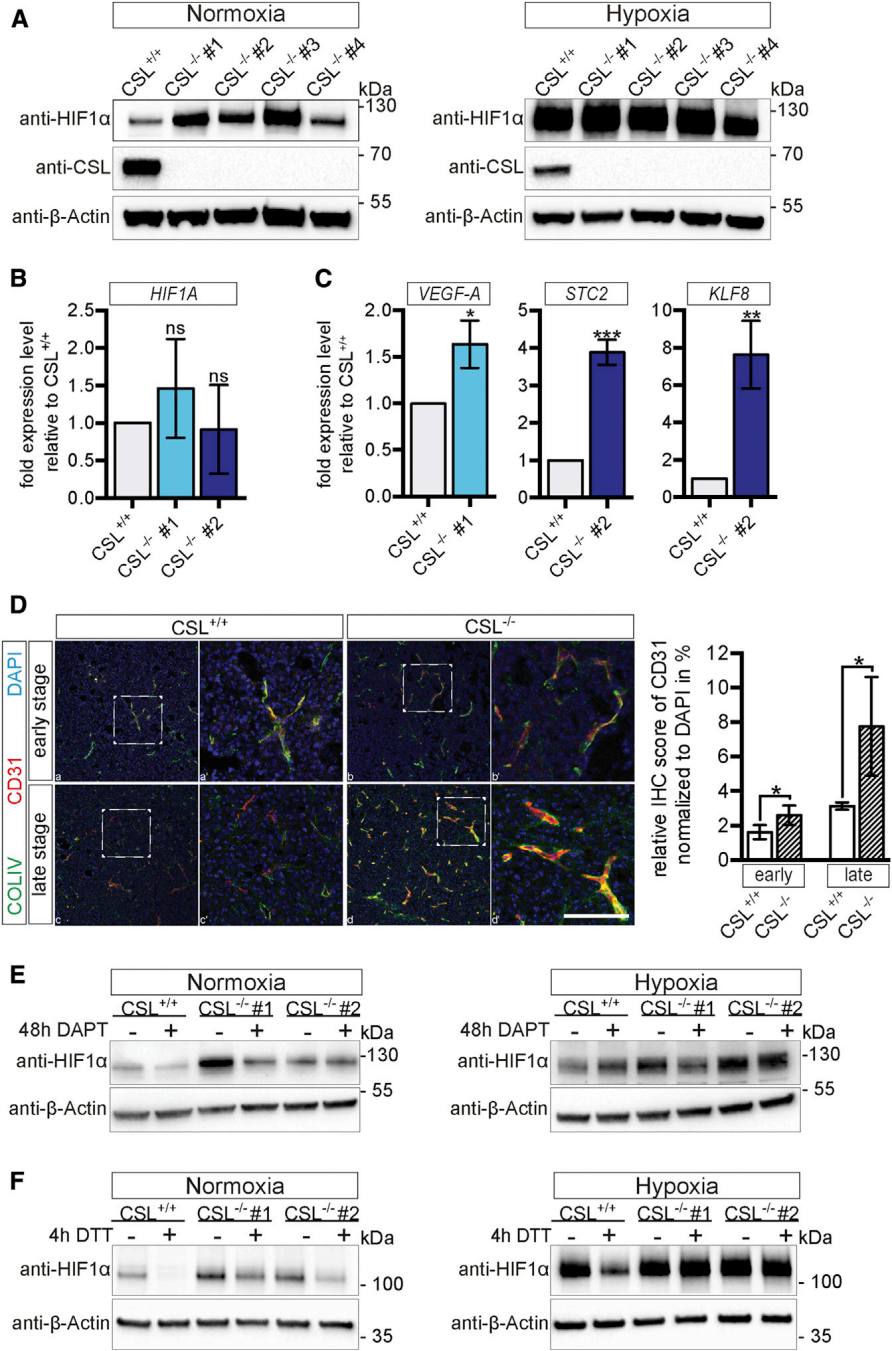
Unleashing Hypoxic Activation and Angiogenic Activity by CSL-Deficient Cells (A) Western blot of HIF1α, CSL, and β-actin (loading control) in control and CSL-deficient MDA-MB-231 cells under normoxic (left) and hypoxic (1% O_2_; right) conditions. (B and C) qPCR analysis of *HIF1α* (B) and *VEGF-A* (clone #1) and *STC2* and *KLF8* (clone #2) (C) mRNA expression in control and CSL^−/−^ cells. mRNA expression level analysis is based on three separate experiments. (D) Representative images of collagen IV (COLIV) and CD31 expression in control and CSL-deficient xenografts. Quantification of the CD31 staining is shown to the right. Signal quantification is based on at least three randomly chosen images from one tumor sample of each kind. Scale bar, 100 μm. (E and F) Western blot of HIF1α and β-actin in control and CSL-deficient MDA-MB-231 cells under normoxic or hypoxic conditions. Cells were cultured in the presence or absence of DTT (E) or DMSO/DAPT (F), as indicated. Data are presented as mean ± SD. ^∗^p ≤ 0.05; ^∗∗^p ≤ 0.01; ^∗∗∗^p ≤ 0.001. ns, not significant.

Hypoxia is an important regulator of tumor growth (Jain, 2014), and hypoxia and Notch signaling intersect in several ways (Gustafsson et al., 2005, Sahlgren et al., 2008, Zheng et al., 2008). This prompted us to assess whether the hypoxic response was altered in CSL^−/−^ cells. Under normoxic conditions the steady-state level of the transcriptional regulator HIF1α is very low, and HIF1α only becomes stabilized during hypoxia. The two MDA-MB-231^CSL−/−^ clones analyzed above as well as two additional clones showed elevated HIF1α protein levels during normoxia compared with the low levels seen in control cells under normoxia (Figure 2A; see Figure S2A for quantification). Reintroduction of CSL into the MDA-MB-231^CSL−/−^ cells abrogated the increase in HIF1α protein levels (Figure S2B). The elevated HIF1α protein levels were a result of post-transcriptional events, as the mRNA levels were similar in CSL^+/+^ and CSL^−/−^ cells (Figure 2B). Activation of hypoxia downstream genes was also observed: in clone #1, *VEGF-A* gene expression was upregulated, whereas the *STC2* and *KLF8* genes showed elevated expression in clone #2 (Figure 2C). In keeping with hypoxia as a potent regulator of tumor vascularization (Rapisarda and Melillo, 2012), vascularization was enhanced around the tumors from CSL^−/−^ cells (data not shown), and collagen IV and CD31 immunostaining (as endothelial markers) in the tumors was elevated (Figure 2D).

Interaction between endogenous Notch1 ICD and HIF1α was observed in the MDA-MB-231^CSL−/−^ cells (Figure S2C), and blocking Notch ICD generation by DAPT reduced the amount of HIF1α in control cells and to a lesser extent in the CSL-deficient clones under normoxic conditions (Figure 2E; see Figure S2D for quantification). Under hypoxic conditions HIF1α levels were not altered in control but reduced in CSL-deficient cells following DAPT treatment (Figure 2E; see Figure S2D for quantification). HIF1α can be stabilized in normoxia and is influenced by nitric oxide and redox potential (Palmer et al., 2000), and we therefore investigated whether the normoxically elevated level of HIF1α in the CSL^−/−^ cells was susceptible to the reducing agent DTT. Treatment by DTT resulted in a decrease in HIF1α in CSL^−/−^ cells, as well as in the low level of HIF1α in control cells, during normoxia, whereas HIF1α was largely unresponsive in the hypoxic CSL-deficient cells (Figure 2F; see Figure S2E for quantification). In conclusion, these data show that HIF1α levels are regulated by CSL.

### CSL-Deficient Cells Acquire a Polyploid Giant-Cell Phenotype and a Mitosis Defect

**Figure 3:**
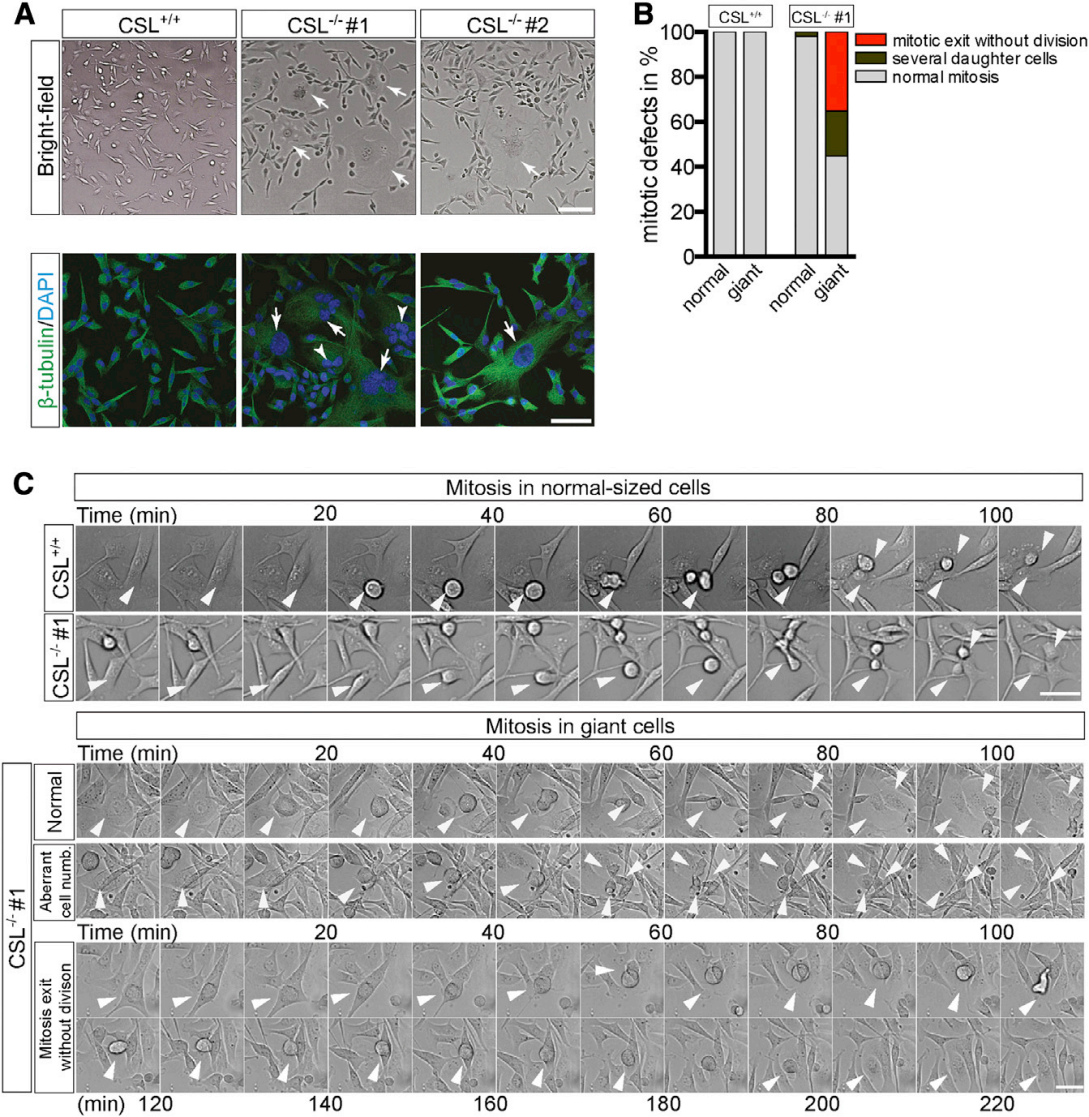
Acquisition of a Polyploid Giant-Cell Phenotype in the CSL-Deficient Cells (A) Brightfield images of CSL^+/+^ and CSL^−/−^ cells (upper panel) and high-magnification view of CSL^+/+^ and CSL^−/−^ cells stained with DAPI and β-tubulin (lower panel). Giant cells are marked with arrows and polyploid cells are marked with arrowheads. (B) Proportion of cells without mitotic exit division and cells generating several daughter cells among CSL^+/+^ and CSL^−/−^ cells. At least 50 cells of each clone were analyzed. (C) Time-lapse images of control and CSL-deficient cells. The white arrowheads denote cells analyzed by time-lapse imaging. Scale bars: 100 μm (A upper), 50 μm (A lower), and 50 μm (C).

Both MDA-MB-231^CSL−/−^ clones were morphologically heterogeneous and presented subcellular populations with a cellular morphology that was distinct from the control cells when cultured in vitro. The phenotype was characterized by cells having a large volume and containing either a giant nucleus or a fragmented polyploid nucleus, and the giant cells were frequently surrounded by small-sized cells (Figures 3A and 3B). To determine the origin of the giant-cell phenotype, we monitored control and CSL^−/−^ cells by time-lapse live-cell microscopy (Figure 3C). Single-cell analysis of mitotic progression in both giant and normal-sized cells revealed that a large proportion of CSL^−/−^ cells presented aberrant mitosis, with cells dividing into multiple daughter cells or by exiting mitosis without dividing into two daughter cells (Figures 3B and 3C, lower panel; videos in Figure S3A). The CSL^−/−^ cells (clone #2) displayed a decreased proliferation rate in vitro, and reintroduction of CSL restored the proliferation rate observed in control cells (Figures S3B and S3C). In sum, these observations show that loss of CSL affects cell morphology and leads to a mitotic defect.

### A Notch-Independent Transcriptional Signature in the CSL-Deficient Cells

**Figure 4:**
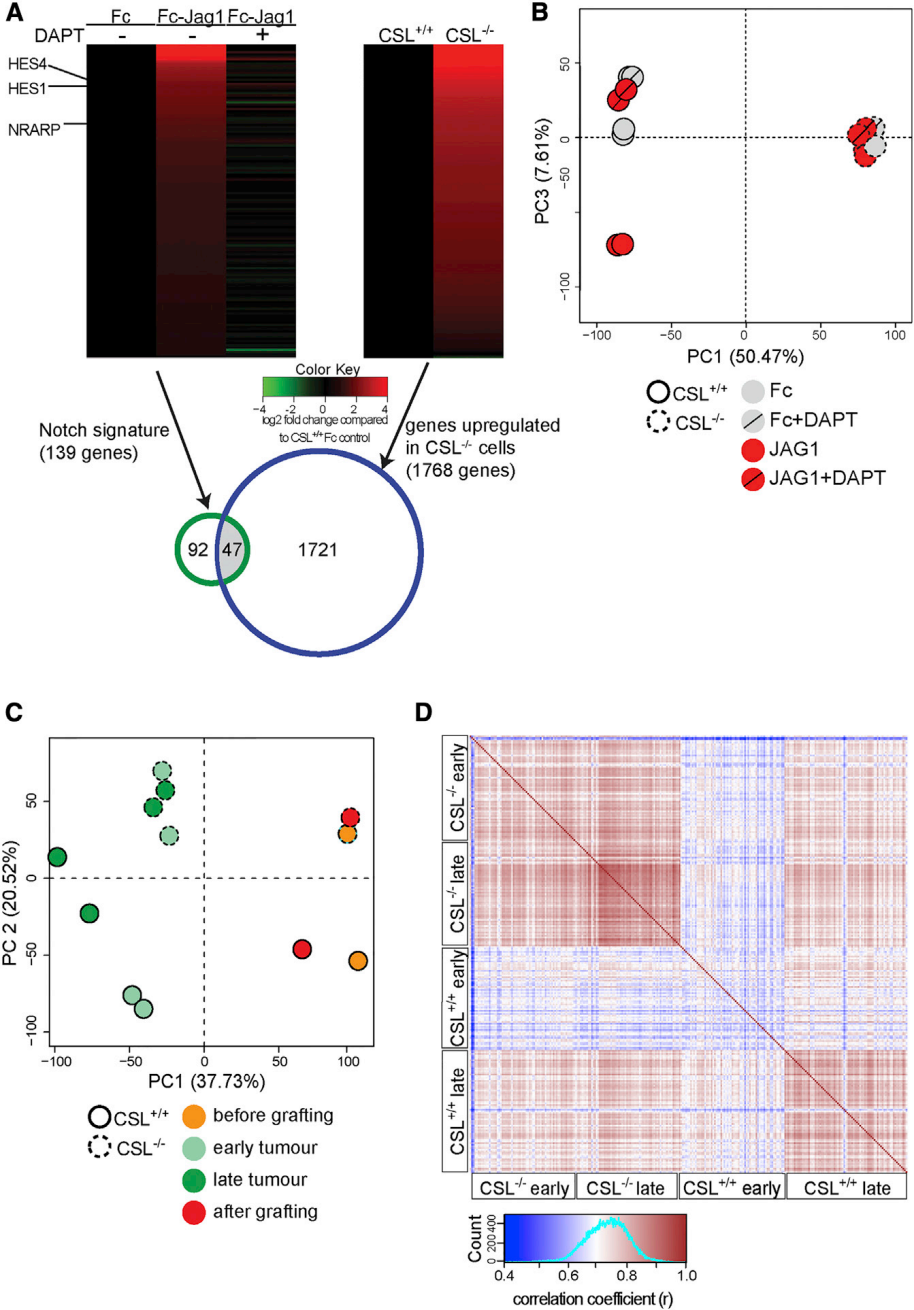
A Notch-Independent Transcriptional Signature in the CSL-Deficient Cells (A) (Left) Heatmap of the 139 genes that constitute the Notch signature, i.e., genes which are upregulated by ligand (Jag1) stimulation and where gene expression is abrogated by DAPT. (Right) Heatmap of the 1,768 genes that were upregulated in CSL-deficient cells compared with control cells. Lower panel: Venn diagram showing the comparison of the Notch signature genes and genes upregulated in CSL-deficient cells. (B) PCA of genome-wide transcriptome analysis for CSL^+/+^ and CSL^−/−^ cells during ligand activation (Jag1) and inhibition by DAPT (n = 2 for each treatment). (C) PCA of genome-wide transcriptome analysis for CSL^+/+^ and CSL^−/−^ cells during in vitro culturing prior to tumor xenografting, the xenograft tumors, and when returned to in vitro culture after xenografting, as indicated. (D) Correlation plot of single-cell RNA-seq analysis from CSL^+/+^ and CSL^−/−^ cells in tumors from early (3 weeks) and late (5 weeks) stages.

We next assessed the transcriptional consequences of CSL deficiency, i.e., whether loss of CSL resulted in only derepression of a Notch transcriptional signature or affected a larger non-Notch-dependent gene set. The Notch transcriptional signature was identified as genes upregulated by ligand activation and where the ligand-induced upregulation was abrogated by blocking Notch receptor cleavage using DAPT. RNA-seq analysis revealed 139 genes that were ligand-activated and sensitive to DAPT, and which we denote the Notch signature (Figure 4A). This gene set contained a number of well-established Notch downstream targets, such as *HES1*, *HES4*, and *NRARP* (Figure 4A). We next compared the transcriptomes of MDA-MB-231^CSL−/−^ and control cells, and 1,768 genes were upregulated in the CSL-deficient cells (Figure 4A). GSEA analysis revealed that this gene set was enriched for genes associated with KRAS and TNFα signaling or involved in angiogenesis, G2M checkpoint or apical junctions, and epithelial-to-mesenchymal transition. In line with this, MMP1 was also significantly upregulated in CSL^−/−^ cells (Figure S4A). A comparison between the 1,768 genes and the 139 genes in the Notch signature revealed that only 47 genes were common to both categories (Figure 4A). The limited overlap was corroborated by a principal component analysis (PCA) showing that CSL^−/−^ cells clustered quite distinctly from control cells, and that ligand activation and DAPT treatment had quite limited effects on the transcriptomes in the CSL^−/−^ cells, whereas the effect was more profound in the control cells (Figure 4B).

To assess how CSL deficiency affected the transition from in vitro culture to the in vivo tumor situation, we compared the transcriptome from in vitro culturing with that from tumors at early and late stages after xenografting, using the S^3^ technology to bioinformatically sort out the tumor (human) from the stromal (mouse) transcripts (Chivukula et al., 2015). PCA revealed that the in vitro transcriptomes from MDA-MB-231^CSL−/−^ and control cells were quite distinct and that the differences were maintained in the tumor situation (Figure 4C). Interestingly, when tumor cells were excised and returned to in vitro culture the transcriptomes largely reverted back to more closely resemble the respective in vitro transcriptomal profile observed prior to transplantation (Figure 4C). Finally, single-cell transcriptome analysis showed that MDA-MB-231^CSL−/−^-derived tumors were more homogeneous than MDA-MB-231^CSL+/+^-derived tumors, and cellular homogeneity further increased at the later tumor stage (Figure 4D). In sum, these data suggest that CSL transcriptionally controls a number of genes that are not part of a core Notch signature.

## Discussion

CSL serves as the central node in canonical Notch signaling by transmitting signaling from all Notch receptors upon ligand activation. In this work, we report that genetic ablation of CSL in breast tumor cells leads to enhanced tumor growth after transplantation into mammary fat pads in mice, an unexpected finding given that blocking of Notch at the receptor level reduces and activation of Notch promotes tumor growth (Bolós et al., 2013, Suman et al., 2013).

The genome-wide transcriptome data support the view that CSL does not merely mediate Notch signaling, as the set of genes upregulated by CSL ablation was considerably larger than the Notch signature in the MDA-MB-231 cells. This conclusion differs from a recent report, which used small hairpin RNA to knock down CSL expression in MDA-MB-231 cells (Kulic et al., 2014). While Kulic et al. (2014), like us, showed that reduced CSL expression in MDA-MB-231 cells promotes tumor growth, they argue that the observed phenotype was linked to derepression of Notch-activated genes. This notion, however, was based on gene-expression analysis of a set of only 170 genes that were on theoretical grounds considered to be Notch responsive (Kulic et al., 2014). Only five of the 170 genes, however, were among the 1,768 genes upregulated in the CSL^−/−^ cells and only two genes were common to the 139 genes in our Notch signature (Figures S4B and S4C). The notion that CSL has Notch-independent functions is in line with the fact that CSL interacts with a number of proteins that are not linked to Notch signaling, such as CTCF, EBNA3c, interferon regulatory factor 4, and RITA (Collins et al., 2014). One hypothesis to explain the large Notch-independent gene set posits that CSL binds to a larger number of genomic sites, only a subset of which can bind Notch ICD, and loss of CSL would thus lead to derepression also of Notch-independent genes. Recent data, however, support a view whereby CSL is dynamically recruited by Notch ICD and not statically bound as a repressor (Castel et al., 2013, Krejcí and Bray, 2007). An alternative hypothesis is that CSL may not directly repress all genes whose expression is altered, but rather control expression of a smaller set of chromatin modifiers or transcriptional regulators, which in turn reset the chromatin landscape and/or alter gene expression on a broader scale. In support of this view, we noted that expression of a number of such factors, such as Serpin, was upregulated in the CSL-deficient cells. That loss of CSL enhances tumor development is further underlined by a recent study reporting that combined silencing of CSL and p53 in cancer-associated fibroblasts leads to stromal and cancer cell expansion (Procopio et al., 2015).

An unexpected consequence of removing CSL was the unlocking of a hypoxic response during normoxia, manifested by a post-transcriptional elevation of HIF1α protein levels and the upregulation of hypoxia-regulated genes such as *VEGF-A*, *STC2*, and *KLF8*. This is in line with previous reports on normoxic HIF1α protein stabilization (Palmer et al., 2000, Ranasinghe et al., 2015; for review see Kuschel et al., 2012), and adds to the emerging view of a multifaceted interplay between Notch and the cellular hypoxic response (Andersson and Lendahl, 2014, Lendahl et al., 2009). Notch1 ICD interacted with HIF1α, and it is noteworthy that blocking Notch ICD generation reduced the amount of HIF1α, raising the intriguing possibility that Notch ICD in some way plays a role in the observed HIF1α stabilization. Furthermore, the HIF1α protein levels in the CSL-deficient cells were reduced by DTT treatment, suggesting a role for redox potential, possibly linked to nitrosylation of HIF1α (Palmer et al., 2000) or destabilization of the ODD domain. The unleashing of a hypoxic response during normoxia may also be linked to the acquisition of aberrant cell morphology, a phenotype strongly reminiscent of the recently described polyploid giant cancer cell (PGCC) phenotype (Zhang et al., 2014). Interestingly, PGCC cells, which are endowed with cancer stem cell-like properties, were recently identified in a number of tumor contexts in response to hypoxia or chemical induction of the hypoxic response by CoCl_2_ (Zhang et al., 2014), suggesting that loss of CSL may lead to a PGCC-like state via upregulation of HIF1α. As PGCC cells are endowed with reduced proliferative rate in vitro combined with accelerated tumor growth capacity (Zhang et al., 2014), the induction of HIF1α protein levels in the CSL-deficient cells may underlie their enhanced growth rates, invasive capacity, and accelerated tumor growth.

In conclusion, the data in this report provide evidence for a role for CSL in controlling the cellular hypoxic response and cell cycle/cytokinesis as well as tumor growth. The data also indicate that CSL acts beyond only mediating Notch signaling.

## Experimental Procedures

### CRISPR/Cas9 Genome Editing

Designed guide RNA sequence targeting CSL (5′-AAACATTGTATATATCTGAC-3′) was cloned and ligated to the guide RNA vector (Addgene). Cells were co-transfected with the guide RNA vector and the Cas9 expression vector. Single-cell colonies were isolated and subjected to Western blot and DNA-sequencing analysis.

### Mammary Fat Pad Xenograft

All animal procedures were approved by the Stockholm's North Ethical Committee for Animal Research (permit No N151/14). 1.5 × 10^6^ MDA-MB-231 control or CSL-deficient cells in culture media were orthotopically injected into the left and right fourth inguinal mammary fat pads of 4- to 6-week-old female immunodeficient NOD/SCID mice. Tumor growth and size was measured twice per week using calipers. Tumor volume was calculated according to the formula *L* × *W*^2^. Mice were euthanized at the third and fifth week after transplantation.

### Statistical Analysis

All statistical analyses were calculated by GraphPad Prism (ver. 6). For further details see Supplemental Information.

## Author Contributions

All authors fulfill the ICMJE guidelines for authorship. E.B.B., Y.L.T., Y.P.P., S.L., H.S.C., Q.D., and S.-B.J. performed the biological experiments. Y.L.T. and D.R. performed the bioinformatics part of the study. A.L., X.L., C.S., S.-B.J., and U.L. designed the study. S.-B.J. and U.L. conceived the study and U.L. was the lead writer of the manuscript. All authors critically read the manuscript, approved the final version, and agree to be accountable for all aspects of the work. Shared first authors: E.B.B. (Figures 2 and S2), Y.L.T. (Figures 4 and S4), Y.P.P. (Figures 1D and 1E).

## Accession Numbers

The accession number for the RNA-seq reported in this paper is GEO: GSE77308.

## Supplemental Information

Document S1. Supplemental Experimental Procedures and Figures S1–S4

Table S1. Antibodies and Primers

Table S2. Raw Data

Table S3. Genes

Data S1. Prism Statistics

Document S2. Article plus Supplemental Information

## References

[bib1] Andersson E.R., Lendahl U. (2014). Therapeutic modulation of Notch signalling—are we there yet?. Nat. Rev. Drug Discov..

[bib2] Andersson E.R., Sandberg R., Lendahl U. (2011). Notch signaling: simplicity in design, versatility in function. Development.

[bib3] Bolós V., Mira E., Martínez-Poveda B., Luxán G., Cañamero M., Martínez-A C., Mañes S., de la Pompa J.L. (2013). Notch activation stimulates migration of breast cancer cells and promotes tumor growth. Breast Cancer Res..

[bib4] Borggrefe T., Oswald F. (2014). Keeping notch target genes off: a CSL corepressor caught in the act. Structure.

[bib5] Castel D., Mourikis P., Bartels S.J.J., Brinkman A.B., Tajbakhsh S., Stunnenberg H.G. (2013). Dynamic binding of RBPJ is determined by Notch signaling status. Genes Dev..

[bib6] Chivukula I.V., Ramsköld D., Storvall H., Anderberg C., Jin S., Mamaeva V., Sahlgren C., Pietras K., Sandberg R., Lendahl U. (2015). Decoding breast cancer tissue-stroma interactions using species-specific sequencing. Breast Cancer Res..

[bib7] Collins K.J., Yuan Z., Kovall R. (2014). Structure and function of the CSL-KyoT2 corepressor complex: a negative regulator of Notch signaling. Structure.

[bib8] Conlon R., Reaume G., Rossant J. (1995). Notch1 is required for the coordinate segmentation of somites. Development.

[bib9] Guruharsha K.G., Kankel M.W., Artavanis-Tsakonas S. (2012). The Notch signalling system: recent insights into the complexity of a conserved pathway. Nat. Rev. Genet..

[bib10] Gustafsson M.V., Zheng X., Pereira T., Gradin K., Jin S., Lundkvist J., Ruas J.L., Poellinger L., Lendahl U., Bondesson M. (2005). Hypoxia requires notch signaling to maintain the undifferentiated cell state. Dev. Cell.

[bib11] Hansson E.M., Teixeira A.I., Gustafsson M.V., Dohda T., Chapman G., Meletis K., Muhr J., Lendahl U. (2006). Recording Notch signaling in real time. Dev. Neurosci..

[bib12] Holliday D.L., Speirs V. (2011). Choosing the right cell line for breast cancer research. Breast Cancer Res..

[bib13] Jain R.K. (2014). Antiangiogenesis strategies revisited: from starving tumors to alleviating hypoxia. Cancer Cell.

[bib14] Jin S., Mutvei P., Chivukula I.V., Andersson E.R., Ramsköld D., Sandberg R., Lee K.L., Kronqvist P., Mamaeva V., Ostling P. (2013). Non-canonical Notch signaling activates IL-6/JAK/STAT signaling in breast tumor cells and is controlled by p53 and IKKα/IKKβ. Oncogene.

[bib15] Krejcí A., Bray S. (2007). Notch activation stimulates transient and selective binding of Su(H)/CSL to target enhancers. Genes Dev..

[bib16] Kulic I., Robertson G., Chang L., Baker J.H.E., Lockwood W.W., Mok W., Fuller M., Fournier M., Wong N., Chou V. (2014). Loss of the Notch effector RBPJ promotes tumorigenesis. J. Exp. Med..

[bib17] Kuschel A., Simon P., Tug S. (2012). Functional regulation of HIF-1α under normoxia—is there more than post-translational regulation?. J. Cell. Physiol..

[bib18] Lake R.J., Tsai P.F., Choi I., Won K.J., Fan H.Y. (2014). RBPJ, the major transcriptional effector of notch signaling, remains associated with chromatin throughout mitosis, suggesting a role in mitotic bookmarking. PLoS Genet..

[bib19] Lendahl U., Lee K.L., Yang H., Poellinger L. (2009). Generating specificity and diversity in the transcriptional response to hypoxia. Nat. Rev. Genet..

[bib20] Lowell S., Benchoua A., Heavey B., Smith A.G. (2006). Notch promotes neural lineage entry by pluripotent embryonic stem cells. PLoS Biol..

[bib21] Maekawa Y., Ishifune C., Tsukumo S., Hozumi K., Yagita H., Yasutomo K. (2015). Notch controls the survival of memory CD4+ T cells by regulating glucose uptake. Nat. Med..

[bib22] Mutvei A.P., Fredlund E., Lendahl U. (2015). Frequency and distribution of Notch mutations in tumor cell lines. BMC Cancer.

[bib23] Nam Y., Sliz P., Song L., Aster J.C., Blacklow S.C. (2006). Structural basis for cooperativity in recruitment of MAML coactivators to Notch transcription complexes. Cell.

[bib24] Oka C., Nakano T., Wakeham A., de la Pompa J.L., Mori C., Sakai T., Okazaki S., Kawaichi M., Shiota K., Mak T.W. (1995). Disruption of the mouse RBP-J kappa gene results in early embryonic death. Development.

[bib25] Palmer L., Gaston B., Johns R. (2000). Normoxic stabilization of hypoxia-inducible factor-1 expression and activity: redox-dependent effect of nitrogen oxides. Mol. Pharmacol..

[bib26] Procopio M.-G., Laszlo C., Al Labban D., Kim D.E., Bordignon P., Jo S.-H., Goruppi S., Menietti E., Ostano P., Ala U. (2015). Combined CSL and p53 downregulation promotes cancer-associated fibroblast activation. Nat. Cell. Biol..

[bib27] Ranasinghe W.K.B., Baldwin G.S., Bolton D., Shulkes A., Ischia J., Patel O. (2015). HIF1α expression under normoxia in prostate cancer—which pathways to target?. J. Urol..

[bib28] Rapisarda A., Melillo G. (2012). Overcoming disappointing results with antiangiogenic therapy by targeting hypoxia. Nat. Rev. Clin. Oncol..

[bib29] Robinson D.R., Kalyana-Sundaram S., Wu Y.-M., Shankar S., Cao X., Ateeq B., Asangani I., Iyer M., Maher C., Grasso C.S. (2011). Functionally recurrent rearrangements of the MAST kinase and Notch gene families in breast cancer. Nat. Med..

[bib30] Sahlgren C., Gustafsson M.V., Jin S., Poellinger L., Lendahl U. (2008). Notch signaling mediates hypoxia-induced tumor cell migration and invasion. Proc. Natl. Acad. Sci. USA.

[bib31] Schmitt T.M., de Pooter R.F., Gronski M., Cho S.K., Ohashi P.S., Zúñiga-Pflücker J.C. (2004). Induction of T cell development and establishment of T cell competence from embryonic stem cells differentiated in vitro. Nat. Immunol..

[bib32] Suman S., Das T.P., Damodaran C. (2013). Silencing NOTCH signaling causes growth arrest in both breast cancer stem cells and breast cancer cells. Br. J. Cancer.

[bib33] Weng A.P., Ferrando A., Lee W., Morris J.P., Silverman L.B., Sanchez-Irizarry C., Blacklow S.C., Look T., Aster J.C. (2004). Activating mutations of NOTCH1 in human T cell acute lymphoblastic leukemia. Science.

[bib34] Westhoff B., Colaluca I.N., D’Ario G., Donzelli M., Tosoni D., Volorio S., Pelosi G., Spaggiari L., Mazzarol G., Viale G. (2009). Alterations of the Notch pathway in lung cancer. Proc. Natl. Acad. Sci. USA.

[bib35] Wilson J.J., Kovall R. (2006). Crystal structure of the CSL-Notch-Mastermind Ternary complex bound to DNA. Cell.

[bib36] Yin X., Farin H.F., van Es J.H., Clevers H., Langer R., Karp J.M. (2014). Niche-independent high-purity cultures of Lgr5+ intestinal stem cells and their progeny. Nat. Methods.

[bib37] Zhang S., Mercado-Uribe I., Xing Z., Sun B., Kuang J., Liu J. (2014). Generation of cancer stem-like cells through the formation of polyploid giant cancer cells. Oncogene.

[bib38] Zheng X., Linke S., Dias J.M., Zheng X., Gradin K., Wallis T.P., Hamilton B.R., Gustafsson M., Ruas J.L., Wilkins S. (2008). Interaction with factor inhibiting HIF-1 defines an additional mode of cross-coupling between the Notch and hypoxia signaling pathways. Proc. Natl. Acad. Sci. USA.

