# Supplementary material for: Loss of CSL Unlocks a Hypoxic Response and Enhanced Tumor Growth Potential in Breast Cancer Cells
Source: Stem Cell Reports. 2016 Apr 7;6(5):643–51. doi: 10.1016/j.stemcr.2016.03.004 (PMC4939550; doi:10.1016/j.stemcr.2016.03.004)
Supplement: Document S1. Supplemental Experimental Procedures and Figures S1–S4 [file mmc1.pdf]

Stem Cell Reports, Volume *6*

Supplemental Information

Loss of CSL Unlocks a Hypoxic Response and Enhanced Tumor Growth Potential in Breast Cancer Cells

Eike-Benjamin Braune, Yat Long Tsoi, Yee Peng Phoon, Sebastian Landor, Helena Silva Cascales, Daniel Ramsköld, Qiaolin Deng, Arne Lindqvist, Xiaojun Lian, Cecilia Sahlgren, Shao-Bo Jin, and Urban Lendahl

Figure S1

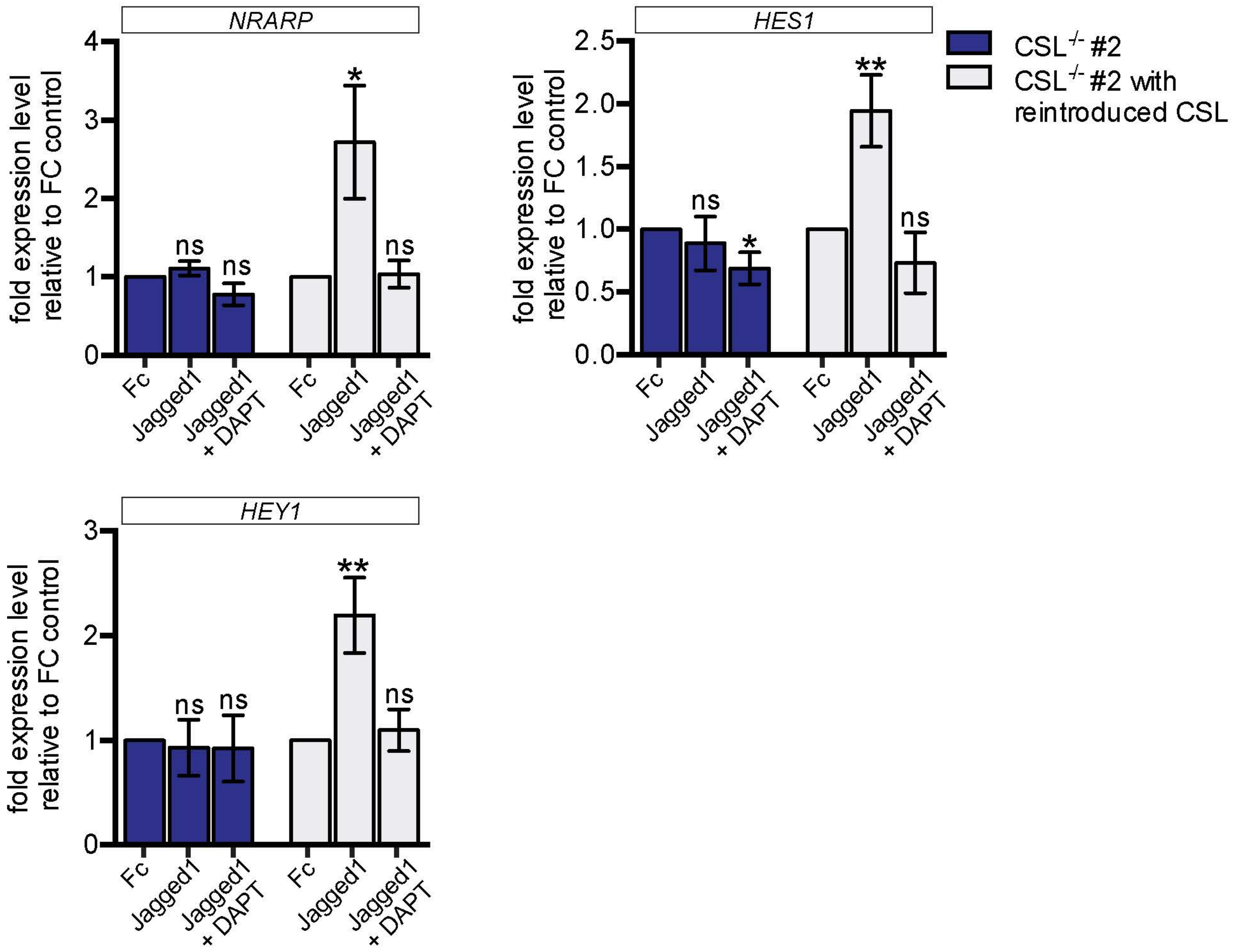
NRARP
HES1
HEY1
fold expression level relative to FC control
CSL-/- #2
CSL-/- #2 with reintroduced CSL
ns
*
**
Fc
Jagged1
Jagged1 + DAPT
0
1
2
3
4
0.0
0.5
1.0
1.5
2.0
2.5

Figure S2

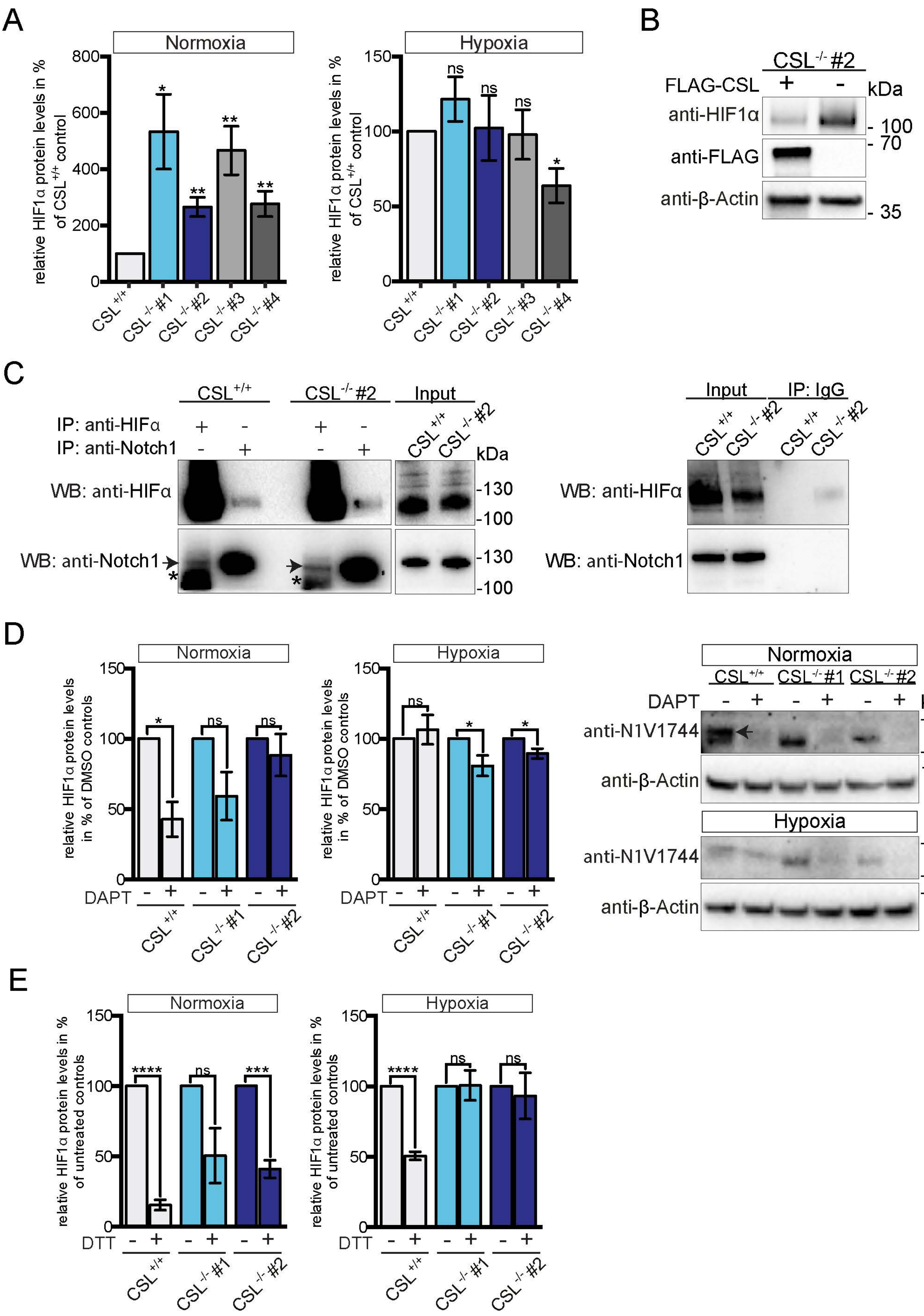
A
Normoxia
Hypoxia
relative HIF1α protein levels in % of CSL+/+ control
CSL+/+
CSL-/- #1
CSL-/- #2
CSL-/- #3
CSL-/- #4
B
CSL-/- #2
FLAG-CSL
anti-HIF1α
anti-FLAG
anti-β-Actin
kDa
C
CSL+/+
Input
IP: anti-HIFα
IP: anti-Notch1
WB: anti-HIFα
WB: anti-Notch1
IP: IgG
D
Normoxia
Hypoxia
relative HIF1α protein levels in % of DMSO controls
DAPT
anti-N1V1744
anti-β-Actin
E
relative HIF1α protein levels in % of untreated controls
DTT

Figure S3

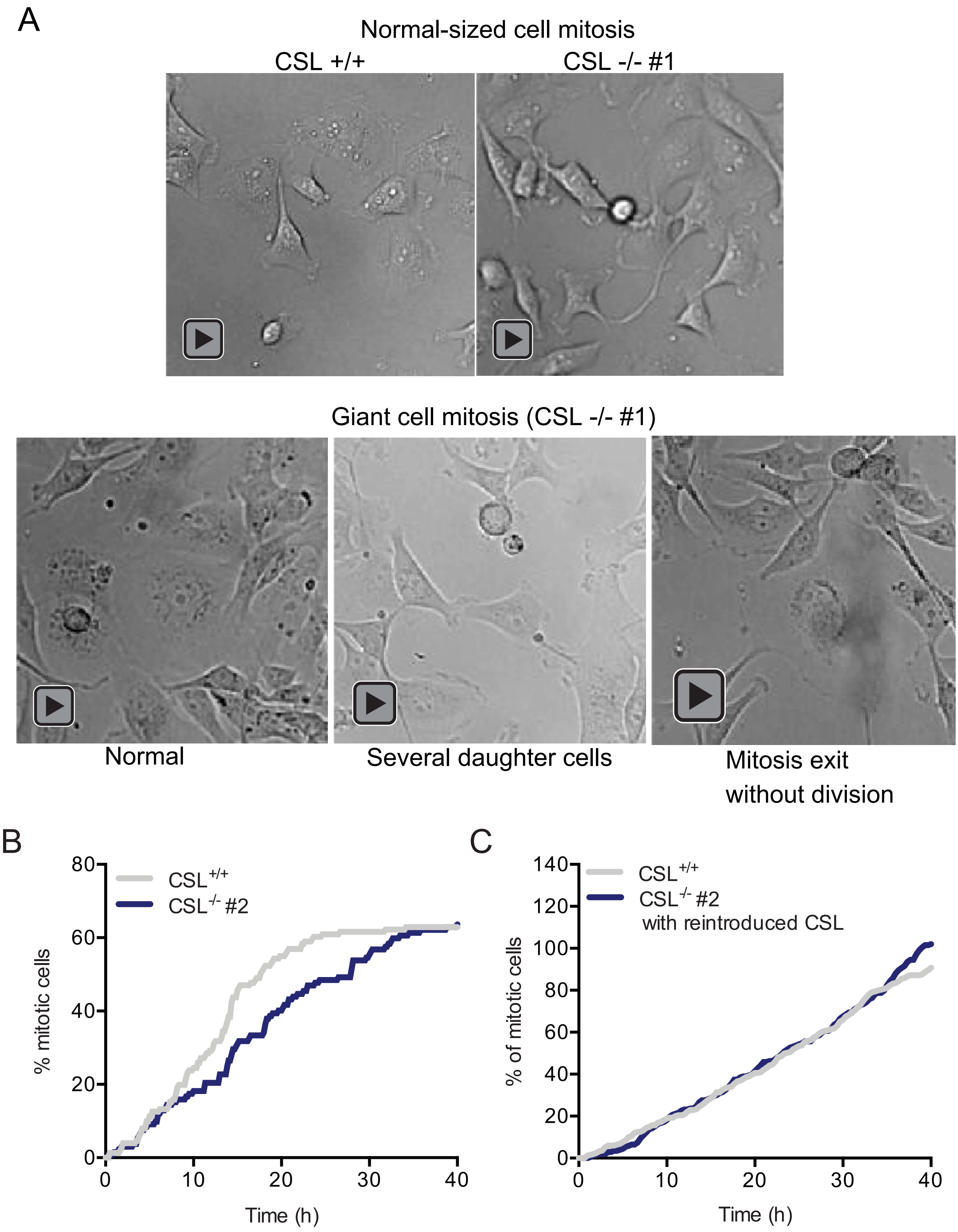
A
Normal-sized cell mitosis
CSL +/+
CSL -/- #1
Giant cell mitosis (CSL -/- #1)
Normal
Several daughter cells
Mitosis exit
without division
B
CSL+/+
CSL-/- #2
% mitotic cells
Time (h)
0
10
20
30
40
0
20
40
60
80
C
CSL+/+
CSL-/- #2
with reintroduced CSL
% of mitotic cells
Time (h)
0
20
40
60
80
100
120
140

Figure S4

A

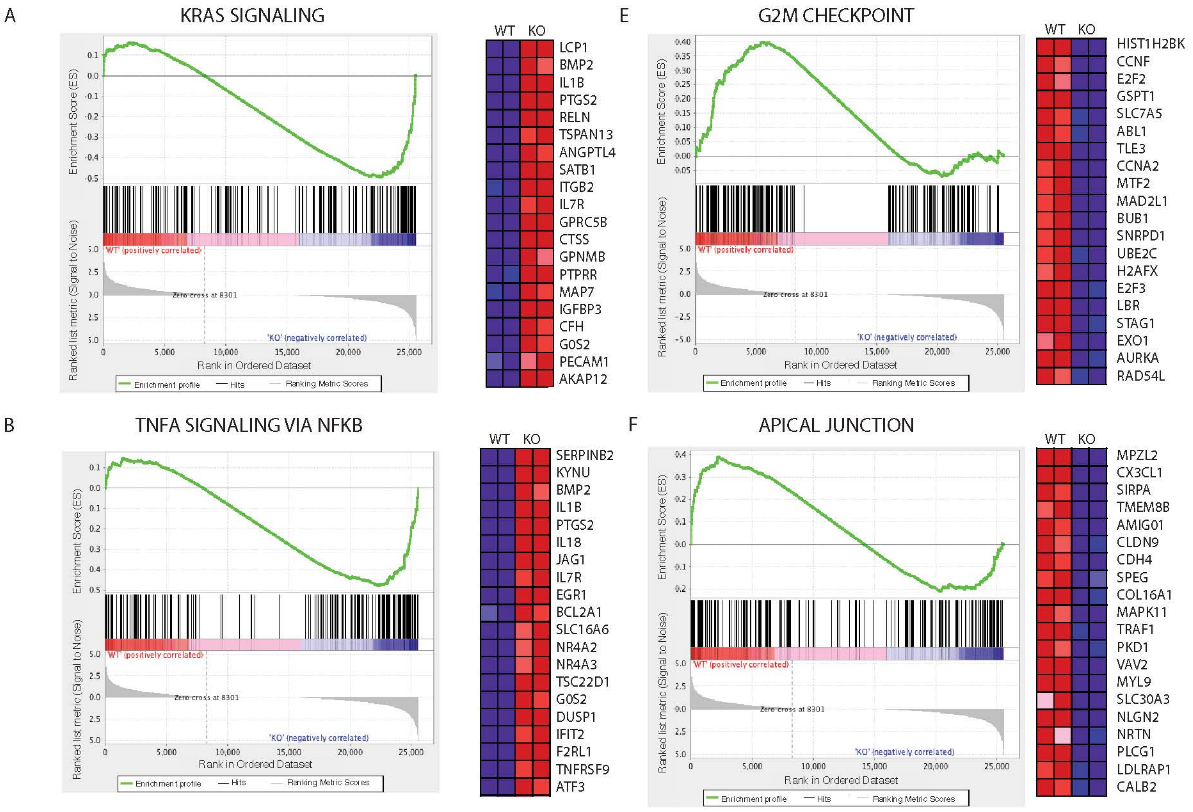

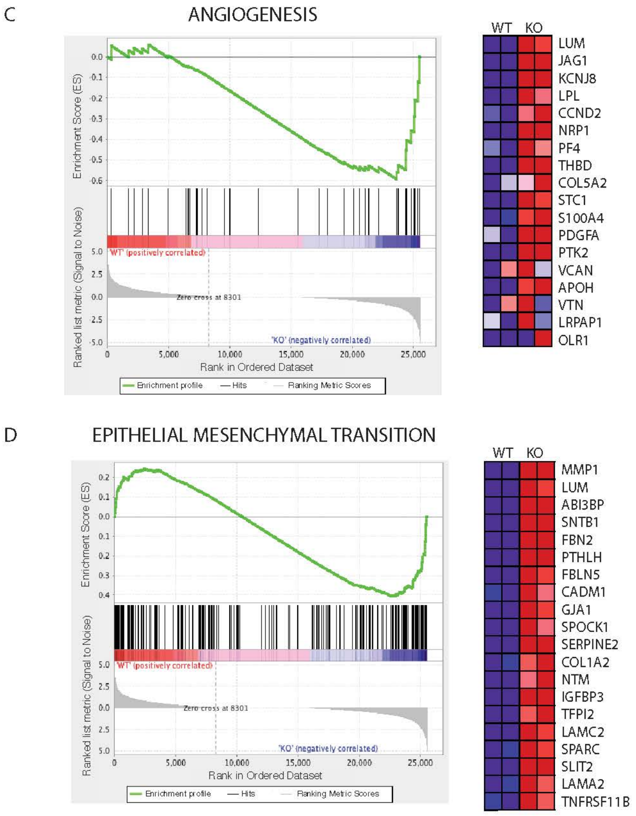

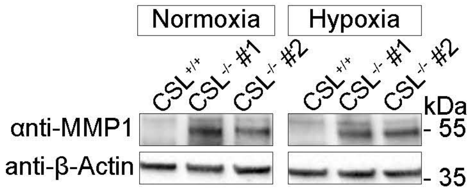

B

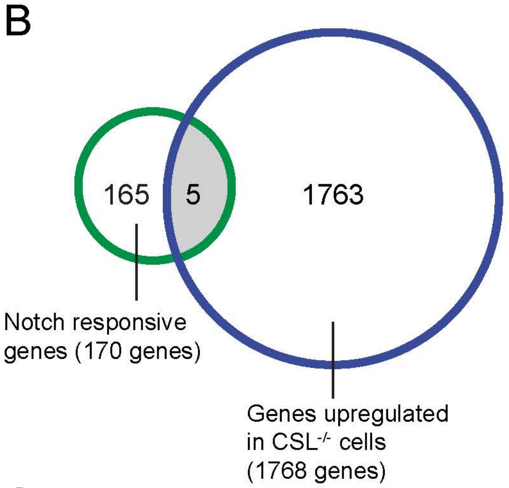

C

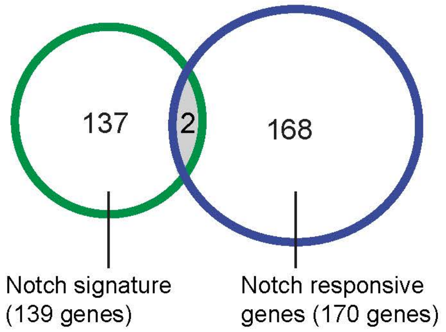

Supplemental Figure legends

Figure S1. Quantitative PCR analysis of *NRARP, HES1* and *HEY1* mRNA expression in $CSL^{-/-}$ #2 cells and in $CSL^{-/-}$ #2 expressing reintroduced FLAG-CSL after activation of Notch (Jagged1) or inhibition of Notch receptor cleavage (Jagged1 + DAPT). mRNA expression level analysis is based on three separate experiments. Data are presented as mean ± SD. * $P \leq 0.05$; ** $P \leq 0.01$.

Figure S2. (A) Quantification of Western blots presented in Figure 2A. **(B)** Western blot of HIFα, FLAG and β-actin (loading control) of lysates from CSL-deficient cells (clone #2) and cells expressing reintroduced FLAG-CSL. **(C)** Immunoprecipitation of endogenous Notch1 or HIFα from control or CSL-deficient cells. Immunoblotting was performed with Notch1 and Hifa antibodies, as indicated. The arrow denotes the Notch1 ICD band and the asterisk (*) denotes a band of unknown nature. **(D)** (left) Quantification of Western blots presented in Figure 2E. (right) Western blot of cleaved Notch1 and β-actin (loading control) in control and CSL-deficient MDA-MB-231 cells under normoxic (top) and hypoxic conditions (bottom) with and without DAPT treatment. The arrow denotes a band of unknown nature that is uniquely present in $CSL^{+/+}$ cells. **(E)** Quantification of Western blots shown in Figure 2F. All graphs presenting quantification of protein levels are based on at least three independent experiments. Data are presented as mean ± SEM. * $P \leq 0.05$; ** $P \leq 0.01$; *** $P \leq 0.001$; **** $P \leq 0.0001$.

Figure S3. (A) Live cell imaging experiments showing cell division and proliferation of control and CSL-deficient cells. **(B)** Graph showing the cumulative mitotic entry of at least 100 cells per condition. The cell cycle time for $CSL^{+/+}$ cells is approximately 30h, the cell cycle time for $CSL^{-/-}$ #2 cells is approximately 50-55h. **(C)** Graph showing the cumulative mitotic entry of control and $CSL^{-/-}$ #2 cells expressing reintroduced FLAG-CSL.

Figure S4. (A) GSEA analysis of $CSL^{+/+}$ and $CSL^{-/-}$ cells. (bottom) Western blot of MMP1 and β-actin (loading control) protein levels in control and CSL-deficient MDA-MB-231 cells under normoxic and hypoxic conditions. Note that MMP1 is only barely detectable in control cells.Venn diagrams: **(B)** the 1768 genes upregulated in the CSL-deficient cells compared to the set of 170 Notch-responsive genes defined by Kulic et al., (2014); **(C)** the 139 genes defined as the Notch signature in this study to the 170 genes defined by Kulic et al., (2014).

Supplemental Experimental Procedures

Cell culturing and transfections

MDA-MB-231 were purchased from American Type Culture Collection (ATCC) and maintained in Dulbecco's Modified Eagle Medium (DMEM) with 10% fetal bovine serum (FBS) and 1% penicillin-streptomycin (Life Techonologies). Cells were transfected using lipofectamine 2000 (Life Technologies) according to the manufacturer's protocols. To block Notch signaling cells were treated with 10 µM DAPT overnight. To activate Notch signaling cells were mechanical dissociated and seeded on 12 well plate precoated with control Fc or Jagged1-Fc (Jag) fragment for 7h.

Genomic DNA purification and sequencing

Genomic DNA from MDA-MB-231 CSL-deficient cells was isolated and purified using the GeneJET Genomic DNA Purification Kit (Life technologies). The CSL sequence targeted by the CRISPR constructs was amplified by PCR, using 40 ng of genomic DNA and a KOD DNA Polymerase (Merck Millipore). PCR was performed for 35 cycles, and the purified PCR product was sequenced. PCR primers used are listed in the respective Supplemental Table.

qRT-PCR

For qRT-PCR, 500ng of RNA was reverse-transcribed using *Maxima First Strand cDNA synthesis* kit (Thermo Scientific). qRT-PCR was performed on *Applied Biosystems 7500 Real-Time PCR System* (ABI 7500; Applied Biosystems) and gene expression was detected with Power SYBR Green (Applied Biosystems). Relative gene expression was determined by normalizing to GAPDH mRNA expression. Primers used in qRT-PCR are listed in the respective Supplemental Table.

Western blot

MDA-MB-231 control and CSL-deficient cells were cultured on a 100 mm culture dishes to 90% confluence and subjected to hypoxic conditions (1% oxygen) for 4-5h or left untreated in normoxic conditions. The cells were washed in ice-cold PBS and subsequently lysed in sample buffer [62.5 mM Tris-HCl, pH 6.8, 25% glycerol, 2% SDS, 5% β-mercaptoethanol, 0.01% bromphenolblue], heated for 10 min to 95°C and either loaded on Mini-Protean Tris-Glycine TGX gradient gels (Biorad) or stored at -80°C. Protein lysates were separated on Mini-Protean Tris-Glycine TGX gradient gels (Bio-rad). After electrophoresis, gels were placed directly onto a Trans-Blot Turbo PVDF membrane transfer pack and transferred using the "HIGH MW" protocol (Bio-Rad). Subsequently, membranes were blocked for 1h at RT using StartingBlock (PBS) buffer (Thermo Scientific) supplemented with 0.1% Tween20 (Sigma- Aldrich). Detection of antibody binding was performed by using Clarity Western ECL-Substrate (Bio-rad). All antibodies used are listed in the respective Supplemental Table. Proteins expression levels were quantified by using ImageJ64 (Ver. 1.48) with beta-Actin for normalization. For signal quantification only non-saturated bands were measured. All calculations are based on a minimum of three independent western blots.

Antibodies, immunohistochemistry and immunocytochemistry

Tumor samples were fixed with 4% paraformaldehyde at 4°C overnight, cytoprotected with 30% sucrose at 4°C overnight, embedded in OCT compound (Tissue-Tek) and sectioned at 8 µm. Sections were blocked in blocking solution (10% normal donkey serum, 1% bovine serum albumin (BSA) and 1% Triton-X (Sigma-Aldrich) in PBS) at room temperature for 1h and incubated with primary antibody diluted in blocking solution at 4°C overnight. Slides were washed with PBS three times five min each and incubated with fluorescence secondary antibody (Alexa Fluorophore) in blocking solution at room temperature for 1h. Slides were washed with PBS for five min three times and mounted with *Vectashield Antifade Mounting Medium with DAPI* (Vector Labs). Confocal images were taken with a Zeiss LSM 700 (Carl Zeiss). All antibodies used are listed in the respective Supplemental Table.

Hematoxylin and eosin (H&E) staining

Cryosection slides of 8 µm thickness were rehydrated in PBS for five min, stained with Gill's hematoxylin (Sigma-Aldrich) diluted 1:1 in PBS for five min and washed with running water for five min. Slides were then

stained with Eosin (Sigma-Aldrich) for 1.5 min, wash briefly in water, dehydrated in a series of dilution (70%, 95%, 95%, 100%, 100% ethanol, xylene, xylene) and mounted with *Organo/Limonene Mount* (Sigma-Aldrich).

Cellular hypoxia and γ-secretase inhibition
MDA-MB-231 control and CSL-deficient cells were cultured on 100 mm cell culture dishes to ca 70% confluence and subjected to hypoxic conditions (1% oxygen) for 48h or left untreated in normoxic conditions. During the incubation the cells were treated with 10 µM DAPT or DMSO as control. After 48h cells were harvested and whole cell lysates were prepared as described above.

Cellular hypoxia and DTT treatment
MDA-MB-231 control and CSL-deficient cells were cultured on 100 mm cell culture dishes to 95% confluence and subjected to hypoxic conditions (1% oxygen) for 4h or left untreated in normoxic conditions. During the incubation the cells were treated with 100 µM DTT or left untreated. After 4h cells were harvested and whole cell lysates were prepared as described above.

HIF1a-Notch1 Immunoprecipitation
MDA-MB-231 control and CSL-deficient cells were cultured on 100 mm cell culture dishes to 90% confluence and subjected to hypoxic conditions (1% oxygen) for 4h or left untreated in normoxic conditions. The cells were washed twice with ice-cold PBS, fixed with 1% PFA for 10 min at room temperature and subsequently incubated with 100 mM Glycine added directly to the medium for five min at room temperature to quench the reaction. The cells were then lysed in ice-cold lysis buffer [20 mM Tris-HCL pH 8, 125 mM NaCl, 2 mM EDTA, 0.025% deoxycholate (DOC), 0.025% SDS, 0.5% Nonidet-P40] supplemented with protease-inhibitor cocktail (CST) for 30 min at 4°C and sonicated using a *Bioruptor UCD-200* sonication device (Diagenode). In addition lysates were passed through a 23G needle attached to a 1 ml syringe to complete cell lysis. Lysates were clarified by 10 min of centrifugation at 10000 x *g* at 4°C. The supernatant was subsequently incubated with the indicated antibodies (see also Supplemental Table) overnight on an end-to-end rotator at 4°C. Normal rabbit IgG #2729 (CST) was used as control. 30 µl Sepharose-G-beads (GE Healthcare) were added to each immunoprecipitation and incubated for 4h at 4°C on an end-to-end rotator. The beads were washed once in low-salt immune complex wash buffer (upstate) and two times in lysis buffer, 20 min each. Dry beads were mixed with sample buffer [62.5 mM Tris-HCl, pH 6.8, 25% glycerol, 2% SDS, 5% β-mercaptoethanol, 0.01% bromphenolblue], heated for 10 min to 95°C and either loaded on Mini-Protean Tris-Glycine TGX gradient gels (Biorad) or stored at -80°C.

Cell migration and invasion assays
Cell migration was determined *in vitro* by using 8.0 mm pore polycarbonate membrane *Corning transwell* inserts (BD Biosciences) according to the manufacturers' instruction. Cells were starved overnight in serum-free media. Then, 2 x 10^4 cells were seeded into the upper transwell chamber with serum-free media containing DMSO or 10 µM DAPT, while media with 10% FBS was added into the lower chamber as chemoattractant and cells were allowed to migrate over night. Cell invasion was determined *in vitro* by utilizing 8.0 mm pore *BD BioCoat Matrigel Invasion Chambers* (BD Biosciences) according to the manufacturers' instruction: 2 x 10^4 cells were seeded into the upper chamber and allowed to invade through the Matrigel-coated membrane under the influence of 10% FBS containing media at the lower chamber for over night. Cells that had migrated across the membrane in both assays were fixed with 4% paraformaldehyde and stained with DAPI. Excess cells were removed using a cotton swab. For each treatment six randomly chosen images were taken. All cells were imaged using a Zeiss Axiovert A1 (Carl Zeiss). Images were analyzed and counted by CellProfiler Ver. 2.1.1 image analysis software (Broad Institute Inc.) with default settings.

In ovo tumor growth
Fertilized chicken eggs were placed in an egg incubator under rotation at 37°C with 60% humidity on day one of embryonic development. On day three eggs were turned, taken off rotation and punctured with a small hole, then covered with adhesive tape. On day eight the holes were expanded and a small plastic ring (5-6 mm in diameter) was placed on top of the chorioallantoic membrane (CAM). 1-2x10^6 MDA-MB-231 control or CSL-deficient cells were suspended in 1:1 PBS and matrigel for a total volume of 30 µl/egg. The cell suspensions were transplanted inside the plastic ring on the CAM and the eggs were covered with parafilm. On day 13 the tumors

were excised and fixed in 3% PFA for four hours at room temperature after which the tumors were dehydrated with ethanol series of 50%, 70%, 70% for one hour each. The tumors were visualized on a Zeiss SteREO Lumar V12 microscope with 0.8x NeoLumar objective and weighed using an analytical laboratory scale.

Transcriptome analysis and single cell RNA-seq

1. RNA preparation. Tumors with surrounding stromal tissues from xenografts were minced and non-enzymatically digested using cell dissociation enzyme-free PBS-based (Gibco) for 30 minutes at 37°C with occasional pipetting. The dissociated cells were then filtered through a 40 mm nylon mesh strainer. Single cell suspension was obtained by re-suspension in CO_2-independent medium (Gibco) for single cell picking. Single cells of xenografts were manually picked under the microscope as previously described (Picelli et al., 2013). Briefly, a single cell was picked using mouth pipette under 20X magnification in 0.5 ml solution. The picked single cell was released into RNase-free PCR strip tube containing hypotonic Smart-seq RNA lysis buffer consisting of 2.3 ml of 19:1 ratio of 0.4% TritonX-100 and RNase inhibitor (Clontech), 1 ml of 10 mM dNTP (Fermentas), 1 ml of 10 mM oligo-dT primer, and ERCC spike-in (Ambion) at 72°C for three min.

2. RNA-seq library preparation. Total RNA from tumors and cells was purified using the *RNeasy Mini* Kit (QIAGEN), according to the manufacturer's instructions. RNA concentration and RNA integrity number (RIN) were calculated by using the *Agilent RNA 6000 Nano* Kit (Agilent Technologies). Two biological replicates for each RNA sample were processed to cDNA library preparations using the *Illumina TruSeq™ RNA sample preparation* kit (Low-Throughput protocol) according to the manufacturer's protocol. Quantification and quality control of the cDNA libraries were done using the *Agilent DNA 1000* Kit (Agilent Technologies). The libraries were sequenced on an *Illumina HiSeq 2000* system.

3. Reverse transcription and PCR Pre-amplification. Reverse transcription of single cell RNA and PCR pre-amplification were performed according to previously described procedure with the specified conditions (Picelli et al., 2013). Briefly, single cell lysates were denatured at 72°C for three min, and followed by first-strand conversion in a reaction mixture of SuperScript II reverse transcriptase (Invitrogen), RNase inhibitor (Clontech), 5X SuperScript II First-Strand buffer (Invitrogen), DTT (Invitrogen), 5M Betaine (Sigma), MgCl2 (Sigma), and custom locked nucleic acid oligonucleotides. Reverse transcription was performed at 42°C for 90 min, followed by 10 cycles of (50°C for two min, 42°C for two min), and inactivation at 70°C for 15 min.

PCR reactions were performed directly after reverse transcription as described previously with the specified condition (Picelli et al., 2013). Briefly, the total volume of cDNA was added into PCR master mix containing KAPA HiFi HotStart ReadyMix (KAPA Biosystems) and ISPCR primers. The PCR reaction was performed at 98°C three min, then 18 cycles of (98°C for 15s, 67°C for 20s, 72°C for six min), and a final extension at 72°C for five min. Next, PCR products were purified using a 1:1 ratio of AMPure XP beads (Beckman Coulter), with the final elution of 15 ml of EB solution (Qiagen). The library quality and size distribution was checked using a High-Sensitivity DNA chip (Agilent Bioanalyzer).

4. Tagmentation and Final PCR amplification. One nanogram of cDNA was used for tagmentation reaction at 55°C for five min in a 20 ml 5X TAPS buffer, 5 ml PEG solution and 0.5 ml Tn5 transposase (Illumina). Then, 5 ml of 0.5% SDS was directly added into the tagmentation reaction and incubated at room temperature for five min. Final PCR amplification was carried out in a 25 ml of 10mM dNTP, 10 ml of 5X PCR buffer, 1 ml of KAPA polymerase (KAPA Biosystems) and 5 ml Index 1 primers (i7) and 5 ml of Index 2 primers (i5) (Illumina). The reaction was performed at 72°C for three min, 95°C for 30s, and 10 cycles of (95°C for 10s, 55°C for 30s, 72°C for 30s), followed by 72°C for five min and 10°C for infinite. Purification was performed with a 1:1 ratio of AMPure XP beads with an elution of 15 ml and purified samples were quality checked on a High-Sensitivity DNA chip. Next, quantification was carried out using Qubit High-Sensitivity DNA kit (Invitrogen). Finally, libraries were diluted to a final concentration of 2 nM and pooled for sequencing using Illumina HiSeq 2000.

5. Bioinformatics Analysis. Bioinformatics Analysis. Differential gene expression analysis was performed with edgeR package (Robinson et al., 2010). Differentially expressed genes with statistical significance were identified with fold change higher than 1.5 and false discovery rate lower than 0.05. PCA were generated with RPKM of individual samples with FactoMineR package available in Bioconductor. Reads per kilobase per million (rpkm) were calculated for GSEA analysis (Mootha et al., 2003; Subramanian et al., 2005) with the default setting and a false discovery rate at 0.25. For other analysis, tag per millions (TPM) values were defined as (rpkm / total rpkm of a sample * 1000000). To analyze the heterogeneity of the single cell transcriptomic data, 5102 genes with the highest average TPM among all samples or in a single tumor were selected for Pearson's correlation and other analysis. The bioinformatics separation of mouse and human transcriptomes from tumor tissue was carried out as described in Chivukula et al., 2015. All RNA-seq data are available at the Gene Expression Omnibus (GEO) website (Edgar et al., 2002). The assigned accession number is GSE77308.

Live-cell microscopy

For live-cell imaging experiments, 4.000 MDA-MB-231$^{CSL-/-}$ and control cells were seeded in 96-well imaging plates (BD Falcon) 16h before imaging on a Leica DMI6000 Imaging System using a 20x objective. After microscopy, images were processed and analysed using ImageJ.

Statistical analysis

For proliferation and apoptosis analysis (Figure 1F and G, respectively) signals of at least four randomly chosen images from one tumor sample of each kind were counted and compared by unpaired t-test. For chorioallantoic tumor size (Figure 1H) analysis of at least five different tumors of each kind were measured and compared by unpaired t-test. Comparison of invasion and migration (Figure 1I,J) is based on at least three independent experiments and was analyzed by unpaired t-test. HIF1a protein levels (Figure 2A,E and F, Figure S2A,D and E) were compared using unpaired t-test and are based on at least three independent experiments. mRNA expression levels were analyzed using unpaired t-test and are based on three separate experiments (Figure 2B,C). Comparison of vascularisation (Figure 2D) is based on signal quantification of at least three randomly chosen images from one tumor sample of each kind and was analyzed by unpaired t-test.

Supplemental References:

Edgar R., Domrachev M., Lash AE. (2002) Gene Expression Omnibus: NCBI gene expression and hybridization array data repository. Nucleic Acids Res. 30, 207-10

Mootha, V. K., Lindgren, C. M., Eriksson, K.-F., Subramanian, A., Sihag, S., Lehar, J., et al. (2003). PGC-1alpha-responsive genes involved in oxidative phosphorylation are coordinately downregulated in human diabetes. Nat. Genet. *34*, 267–273.

Picelli, S., Björklund, Å. K., Faridani, O. R., Sagasser, S., Winberg, G., & Sandberg, R. (2013). Smart-seq2 for sensitive full-length transcriptome profiling in single cells. Nat. Methods. *10*, 1096–8.

Robinson, M. D., McCarthy, D. J., & Smyth, G. K. (2010). edgeR: a Bioconductor package for differential expression analysis of digital gene expression data. Bioinformatics *26*, 139–140.

Subramanian, A., Tamayo, P., Mootha, V. K., Mukherjee, S., Ebert, B. L., Gillette, M., et al. (2005). Gene set enrichment analysis: a knowledge-based approach for interpreting genome-wide expression profiles. Proc. Natl. Acad. Sci. U. S. A. *102*, 15545–50.
