# Supplementary material for: Loss of CSL Unlocks a Hypoxic Response and Enhanced Tumor Growth Potential in Breast Cancer Cells
Source: Stem Cell Reports. 2016 Apr 7;6(5):643–51. doi: 10.1016/j.stemcr.2016.03.004 (PMC4939550; doi:10.1016/j.stemcr.2016.03.004)
Supplement: Document S2. Article plus Supplemental Information [file mmc6.pdf]

Stem Cell Reports
Report
OPEN ACCESS

Loss of CSL Unlocks a Hypoxic Response and Enhanced Tumor Growth Potential in Breast Cancer Cells

Eike-Benjamin Braune,[1,4] Yat Long Tsoi,[1,4] Yee Peng Phoon,[1,4] Sebastian Landor,[1,2] Helena Silva Cascales,[1] Daniel Ramsköld,[3] Qiaolin Deng,[1] Arne Lindqvist,[1] Xiaojun Lian,[1] Cecilia Sahlgren,[2] Shao-Bo Jin,[1,*] and Urban Lendahl[1,*]
[1]Department of Cell and Molecular Biology, Karolinska Institutet, 17177 Stockholm, Sweden
[2]Turku Centre for Biotechnology, Abo Akademi University and University of Turku, 20520 Turku, Finland
[3]Rheumatology Unit, Department of Medicine Solna, Karolinska Institutet, Karolinska University Hospital, 17176 Stockholm, Sweden
[4]Co-first author
*Correspondence: shaobo.jin@ki.se (S.-B.J.), urban.lendahl@ki.se (U.L.)
http://dx.doi.org/10.1016/j.stemcr.2016.03.004

SUMMARY

Notch signaling is an important regulator of stem cell differentiation. All canonical Notch signaling is transmitted through the DNA-binding protein CSL, and hyperactivated Notch signaling is associated with tumor development; thus it may be anticipated that CSL deficiency should reduce tumor growth. In contrast, we report that genetic removal of CSL in breast tumor cells caused accelerated growth of xenografted tumors. Loss of CSL unleashed a hypoxic response during normoxic conditions, manifested by stabilization of the HIF1α protein and acquisition of a polyploid giant-cell, cancer stem cell-like, phenotype. At the transcriptome level, loss of CSL upregulated more than 1,750 genes and less than 3% of those genes were part of the Notch transcriptional signature. Collectively, this suggests that CSL exerts functions beyond serving as the central node in the Notch signaling cascade and reveals a role for CSL in tumorigenesis and regulation of the cellular hypoxic response.

INTRODUCTION

In most cellular contexts Notch signaling acts as a gatekeeper to differentiation, promoting maintenance of stem or progenitor cell fates (Andersson et al., 2011; Guruharsha et al., 2012). Modulation of Notch signaling is used to control stem or progenitor cell differentiation in vitro, for example toward neural, intestinal, or hematopoietic lineages (Lowell et al., 2006; Schmitt et al., 2004; Yin et al., 2014). Deregulated Notch signaling is increasingly linked to cancer, and Notch receptor mutations are found in, for example, T cell leukemia, non-small cell lung cancer, and breast cancer as well as in several types of tumor cell lines (Mutvei et al., 2015; Robinson et al., 2011; Weng et al., 2004; Westhoff et al., 2009). Notch signaling is also frequently hyperactivated in a range of tumors, including breast cancer (for review see Andersson and Lendahl, 2014).

Notch signaling ensues when transmembrane Notch ligands of the Jagged or Delta-like type interact with Notch receptors on a juxtaposed cell. This results in proteolytic cleavage and liberation of the intracellular domain of the Notch receptor (Notch ICD), which relocates to the cell nucleus and interacts with the DNA-binding protein CSL (also known as RBP-Jk or CBF1), thus making CSL the central node in the signaling cascade for all four Notch receptors (Notch 1–4) (Andersson et al., 2011). In the "Notch off" state, CSL acts as a repressor and binds a number of transcriptional co-repressors, such as SHARP/MINT, KDM5A, and KyoT2 (for review see Borggrefe and Oswald, 2014). In the "Notch on" state, i.e., upon binding to Notch ICD, CSL sheds the co-repressors and instead recruits co-activators, such as p300 and PCAF, converting it to an activator. The interaction between Notch ICD and CSL is stabilized by the MAML protein, and the ternary Notch ICD/MAML/CSL complex induces expression of Notch downstream genes (Nam et al., 2006; Wilson and Kovall, 2006). It has traditionally been assumed that CSL serves as a DNA-bound repressor in the absence of Notch, and in line with this, CSL can bind to DNA in the absence of Notch and remains bound to DNA even during mitosis (Lake et al., 2014). Recent studies, however, provide support for a more dynamic view whereby CSL is recruited to the DNA by Notch ICD (Castel et al., 2013; Krejcí and Bray, 2007).

It is an open question whether CSL only transmits the signal from the Notch receptors or also plays a role in other, non-Notch-related signaling transductions. Gene-targeting experiments show that phenotypes resulting from targeting of Notch ligands or receptors in some situations are phenocopied by targeting of CSL, for example during somitogenesis (Conlon et al., 1995; Oka et al., 1995) or in memory T cells (Maekawa et al., 2015), which is in line with CSL functioning exclusively as the central hub in the Notch signaling cascade (Guruharsha et al., 2012). On the other hand, there are also an increasing number of proteins, such as CTCF, EBNA3c, interferon regulatory factor 4, and RITA (see Collins et al., 2014 and references therein), which are not part of the Notch signaling mechanism but interact

This is an open access article under the CC BY license (http://creativecommons.org/licenses/by/4.0/).

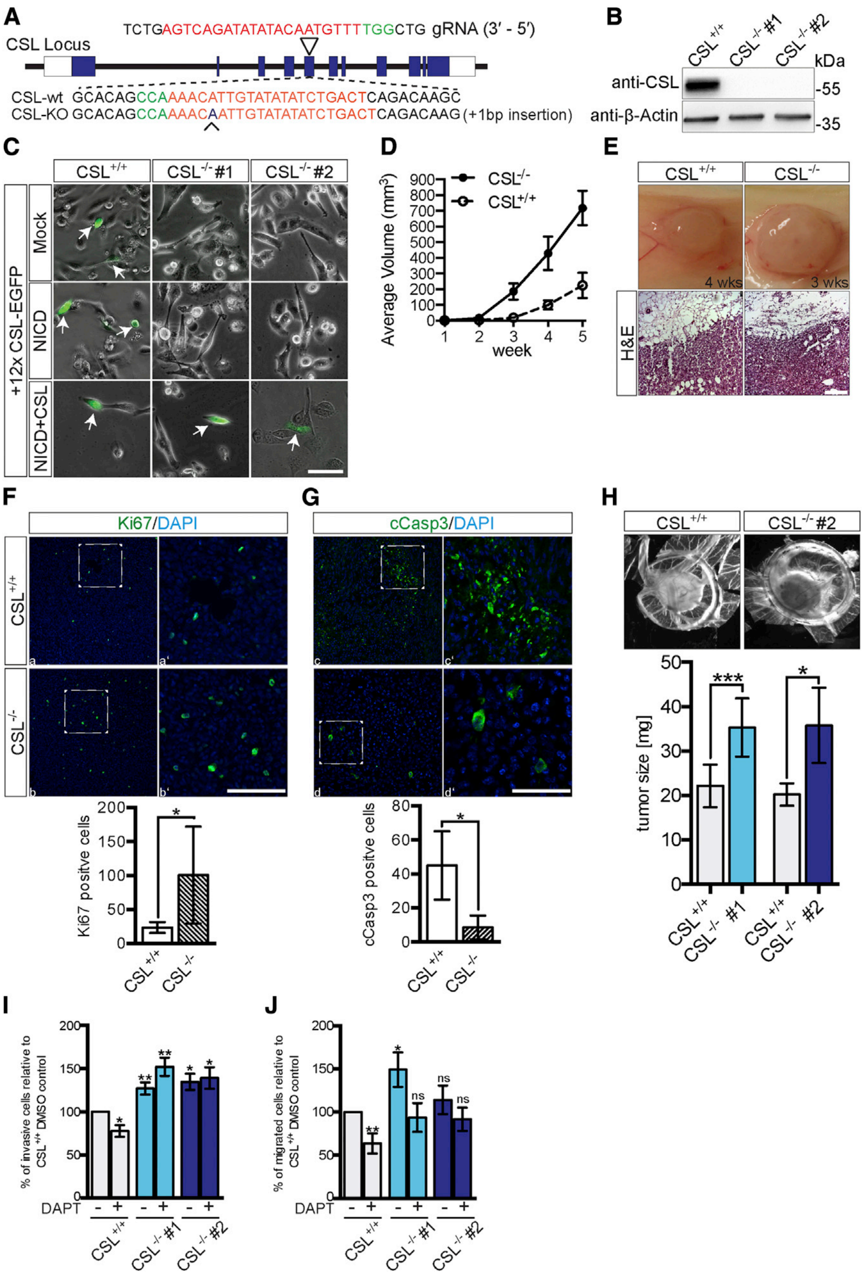

Figure 1. CSL-Deficient Cells Accelerate Tumor Growth In Vivo

(A) Schematic representation of CRISPR/Cas9 targeting of the CSL locus. The triangle points to the targeted exon. Red letters represent the guide RNA sequence and green letters the PAM sequence.

(B) Western blot of CSL and β-actin (loading control) in control ($CSL^{+/+}$) and two clones of CSL-deficient MDA-MB-231 cells ($CSL^{-/-}$).

(C) Notch reporter (12x CSL-EGFP) activity in control and CSL-deficient cells after transfection of 12xCSL-EGFP, Notch1-ICD (NICD), and CSL, as indicated. White arrows indicate cells expressing EGFP.

(D) Average tumor volume at different time points after xenografting $CSL^{+/+}$ or $CSL^{-/-}$ cells. Eight tumors of four mice per group were analyzed.

(E) Representative images and H&E stainings of control and CSL-deficient tumors.

(F and G) Analysis of Ki67 (F) and cleaved Caspase-3 (cCasp3) (G) expression in MDA-MB-$231^{CSL+/+}$ and CSL-deficient tumor sections (enlarged images to the right). At the bottom of each figure, the number of positive cells is quantified. Signals of at least four randomly chosen images from one tumor sample of each kind were counted.

(H) Analysis and quantification of tumor growth in the chick chorioallantoic membrane (CAM) assay for $CSL^{+/+}$ and $CSL^{-/-}$ cells. At least five different tumors of each kind were measured.

(I and J) Invasion and migration assays for $CSL^{+/+}$ and $CSL^{-/-}$ cells. This analysis is based on at least three independent experiments. Data are shown as percent of wild-type MDA-MB-231 DMSO control cells (set to 100%). Data are presented as mean ± SEM. $*p \leq 0.05$; $**p \leq 0.01$; $***p \leq 0.001$. ns, not significant. Scale bars: 100 μm (C), 200 μm (E lower), 100 μm (F and G), and 75 μm (F and G inset).

with CSL, suggesting that CSL has a broader range of actions extending beyond only transmitting Notch signaling.

In this study, we address the question of possible additional roles for CSL and report the unexpected discovery that transplanted breast tumor cells in which CSL was genetically ablated caused rapid tumor growth, a phenotype opposite to blocking Notch function at the receptor level. The phenotype was accompanied by acquisition of a hypoxic response during normoxia and a polyploid giant-cell, cancer stem cell-like, morphology.

RESULTS

Loss of CSL Promotes Tumor Growth In Vivo

To explore the role of CSL in a breast tumor context, we targeted both CSL alleles by CRISPR/Cas9 genome editing in MDA-MB-231 cells (Figure 1A), a breast tumor cell line with active Notch signaling and which promotes tumor growth when transplanted in vivo (Holliday and Speirs, 2011; Jin et al., 2013). In the two independent MDA-MB-$231^{CSL-/-}$ clones selected for further analysis, there was as expected no detectable CSL protein (Figure 1B), and

the activity of a Notch reporter construct (12x CSL-EGFP) (Hansson et al., 2006) was abrogated (Figure 1C). Reintroduction of CSL into the MDA-MB-231$^{CSL-/-}$ cells restored Notch reporter activity (Figure 1C) as well as expression of established Notch downstream genes (Figure S1).

Transplantation of the MDA-MB-231$^{CSL-/-}$ cells into the mammary fat pad in mice resulted in accelerated tumor growth compared with control MDA-MB-231$^{CSL+/+}$ cells. The difference was already noticeable after 3 weeks, and after 5 weeks the tumor volume from the MDA-MB-231$^{CSL-/-}$ cells was 2.8 times larger than in the control cell line (Figures 1D and 1E). Proliferation was increased and apoptosis decreased in the MDA-MB-231$^{CSL-/-}$ tumors as determined by Ki67 (Figure 1F) and cleaved Caspase-3 (Figure 1G) staining, respectively. To assess tumor growth potential in an alternative manner, we cultured both CSL-deficient clones on the chorioallantoic membrane in eggs, and tumor growth was robustly enhanced for both clones (Figure 1H).

In keeping with the tumor data, both CSL$^{-/-}$ clones displayed elevated penetration in a Matrigel invasion assay (Figure 1I). In a transwell migration assay, clone #1 showed enhanced migration whereas migration was not significantly changed in clone #2 (Figure 1J). Treatment with the γ-secretase inhibitor DAPT, which blocks receptor cleavage and thus Notch1 ICD generation, inhibited cell migration and reduced the invasion of CSL$^{+/+}$ but not of CSL-deficient cells (Figures 1I and 1J). In conclusion, these data show that removal of CSL enhances tumor growth in vivo and invasiveness in vitro, and exerts an effect distinct from blockade at the Notch receptor level.

Loss of CSL Unleashes a Hypoxic Response under Normoxic Conditions

Hypoxia is an important regulator of tumor growth (Jain, 2014), and hypoxia and Notch signaling intersect in several ways (Gustafsson et al., 2005; Sahlgren et al., 2008; Zheng et al., 2008). This prompted us to assess whether the hypoxic response was altered in CSL$^{-/-}$ cells. Under normoxic conditions the steady-state level of the transcriptional regulator HIF1α is very low, and HIF1α only becomes stabilized during hypoxia. The two MDA-MB-231$^{CSL-/-}$ clones analyzed above as well as two additional clones showed elevated HIF1α protein levels during normoxia compared with the low levels seen in control cells under normoxia (Figure 2A; see Figure S2A for quantification). Reintroduction of CSL into the MDA-MB-231$^{CSL-/-}$ cells abrogated the increase in HIF1α protein levels (Figure S2B). The elevated HIF1α protein levels were a result of post-transcriptional events, as the mRNA levels were similar in CSL$^{+/+}$ and CSL$^{-/-}$ cells (Figure 2B). Activation of hypoxia downstream genes was also observed: in clone #1, *VEGF-A* gene expression was upregulated, whereas the *STC2* and *KLF8* genes showed elevated expression in clone #2 (Figure 2C). In keeping with hypoxia as a potent regulator of tumor vascularization (Rapisarda and Melillo, 2012), vascularization was enhanced around the tumors from CSL$^{-/-}$ cells (data not shown), and collagen IV and CD31 immunostaining (as endothelial markers) in the tumors was elevated (Figure 2D).

Interaction between endogenous Notch1 ICD and HIF1α was observed in the MDA-MB-231$^{CSL-/-}$ cells (Figure S2C), and blocking Notch ICD generation by DAPT reduced the amount of HIF1α in control cells and to a lesser extent in the CSL-deficient clones under normoxic conditions (Figure 2E; see Figure S2D for quantification). Under hypoxic conditions HIF1α levels were not altered in control but reduced in CSL-deficient cells following DAPT treatment (Figure 2E; see Figure S2D for quantification). HIF1α can be stabilized in normoxia and is influenced by nitric oxide and redox potential (Palmer et al., 2000), and we therefore investigated whether the normoxically elevated level of HIF1α in the CSL$^{-/-}$ cells was susceptible to the reducing agent DTT. Treatment by DTT resulted in a decrease in HIF1α in CSL$^{-/-}$ cells, as well as in the low level of HIF1α in control cells, during normoxia, whereas HIF1α was largely unresponsive in the hypoxic CSL-deficient cells (Figure 2F; see Figure S2E for quantification). In conclusion, these data show that HIF1α levels are regulated by CSL.

CSL-Deficient Cells Acquire a Polyploid Giant-Cell Phenotype and a Mitosis Defect

Both MDA-MB-231$^{CSL-/-}$ clones were morphologically heterogeneous and presented subcellular populations with a cellular morphology that was distinct from the control cells when cultured in vitro. The phenotype was characterized by cells having a large volume and containing either a giant nucleus or a fragmented polyploid nucleus, and the giant cells were frequently surrounded by small-sized cells (Figures 3A and 3B). To determine the origin of the giant-cell phenotype, we monitored control and CSL$^{-/-}$ cells by time-lapse live-cell microscopy (Figure 3C). Single-cell analysis of mitotic progression in both giant and normal-sized cells revealed that a large proportion of CSL$^{-/-}$ cells presented aberrant mitosis, with cells dividing into multiple daughter cells or by exiting mitosis without dividing into two daughter cells (Figures 3B and 3C, lower panel; videos in Figure S3A). The CSL$^{-/-}$ cells (clone #2) displayed a decreased proliferation rate in vitro, and reintroduction of CSL restored the proliferation rate observed in control cells (Figures S3B and S3C). In sum, these observations show that loss of CSL affects cell morphology and leads to a mitotic defect.

A Notch-Independent Transcriptional Signature in the CSL-Deficient Cells

We next assessed the transcriptional consequences of CSL deficiency, i.e., whether loss of CSL resulted in only

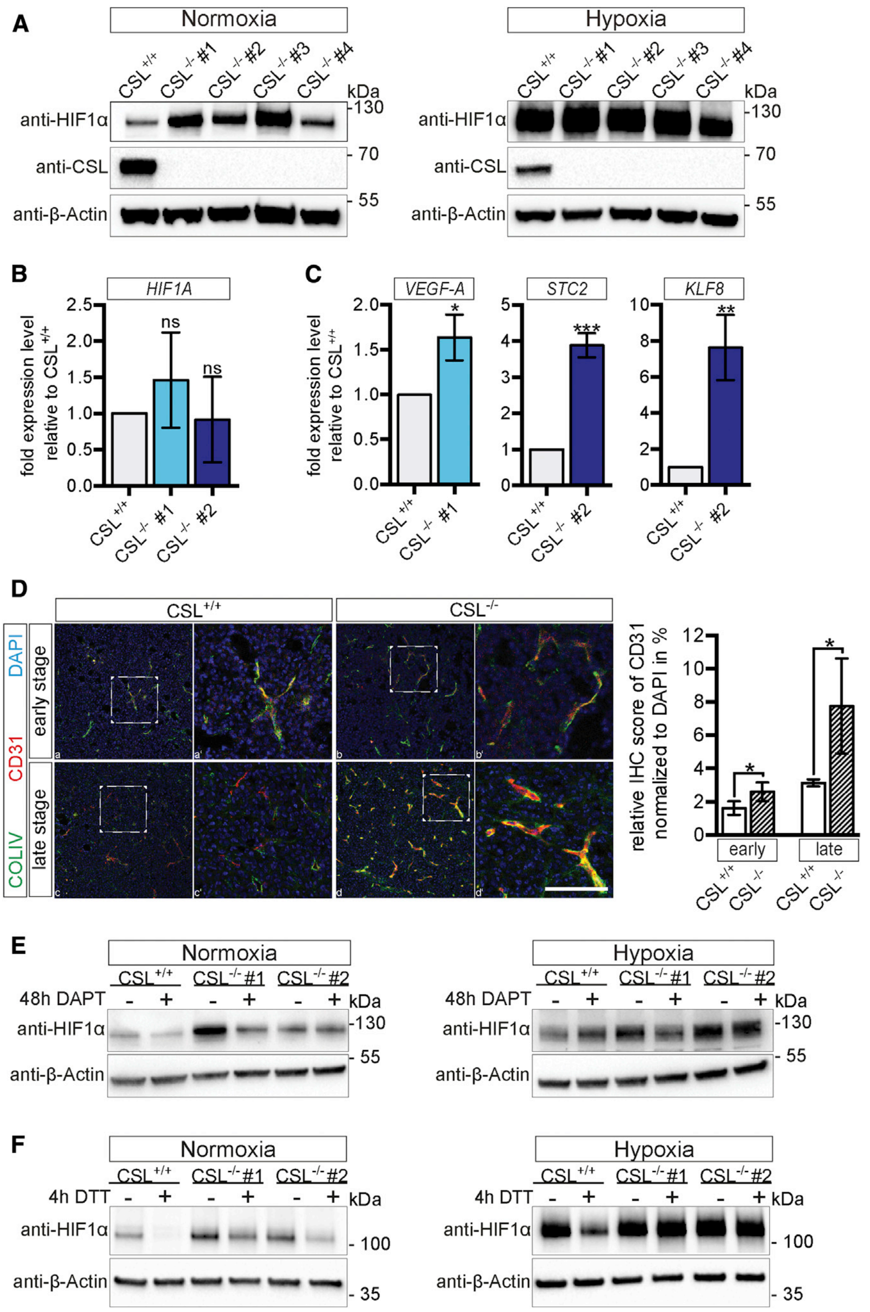

Figure 2. Unleashing Hypoxic Activation and Angiogenic Activity by CSL-Deficient Cells

(A) Western blot of HIF1α, CSL, and β-actin (loading control) in control and CSL-deficient MDA-MB-231 cells under normoxic (left) and hypoxic (1% O_2; right) conditions.

(B and C) qPCR analysis of *HIF1α* (B) and *VEGF-A* (clone #1) and *STC2* and *KLF8* (clone #2) (C) mRNA expression in control and CSL$^{-/-}$ cells. mRNA expression level analysis is based on three separate experiments.

(D) Representative images of collagen IV (COLIV) and CD31 expression in control and CSL-deficient xenografts. Quantification of the CD31 staining is shown to the right. Signal quantification is based on at least three randomly chosen images from one tumor sample of each kind. Scale bar, 100 μm.

(E and F) Western blot of HIF1α and β-actin in control and CSL-deficient MDA-MB-231 cells under normoxic or hypoxic conditions. Cells were cultured in the presence or absence of DTT (E) or DMSO/DAPT (F), as indicated.

Data are presented as mean ± SD. $*p \leq 0.05$; $**p \leq 0.01$; $***p \leq 0.001$. ns, not significant.

derepression of a Notch transcriptional signature or affected a larger non-Notch-dependent gene set. The Notch transcriptional signature was identified as genes upregulated by ligand activation and where the ligand-induced upregulation was abrogated by blocking Notch receptor cleavage using DAPT. RNA-seq analysis revealed 139 genes that were ligand-activated and sensitive to DAPT, and which we denote the Notch signature (Figure 4A). This gene set contained a number of well-established Notch downstream targets, such as *HES1*, *HES4*, and *NRARP* (Figure 4A). We next compared the transcriptomes of MDA-MB-231$^{CSL-/-}$ and control cells, and 1,768 genes were upregulated in the CSL-deficient cells (Figure 4A). GSEA analysis revealed that this gene set was enriched for genes associated with KRAS and TNFα signaling or involved in angiogenesis, G2M checkpoint or apical junctions, and epithelial-to-mesenchymal transition. In line with this, MMP1 was also significantly upregulated in CSL$^{-/-}$ cells (Figure S4A). A comparison between the 1,768 genes and the 139 genes in the Notch signature revealed that only

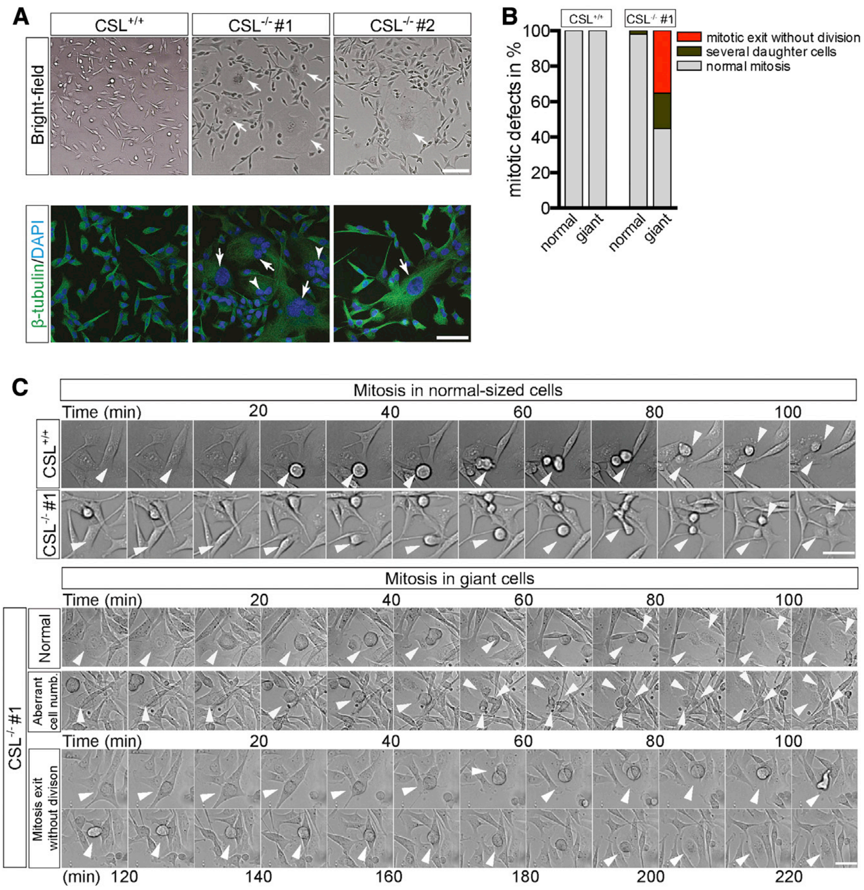

Figure 3. Acquisition of a Polyploid Giant-Cell Phenotype in the CSL-Deficient Cells

(A) Brightfield images of $CSL^{+/+}$ and $CSL^{-/-}$ cells (upper panel) and high-magnification view of $CSL^{+/+}$ and $CSL^{-/-}$ cells stained with DAPI and β-tubulin (lower panel). Giant cells are marked with arrows and polyploid cells are marked with arrowheads.

(B) Proportion of cells without mitotic exit division and cells generating several daughter cells among $CSL^{+/+}$ and $CSL^{-/-}$ cells. At least 50 cells of each clone were analyzed.

(C) Time-lapse images of control and CSL-deficient cells. The white arrowheads denote cells analyzed by time-lapse imaging.

Scale bars: 100 μm (A upper), 50 μm (A lower), and 50 μm (C).

47 genes were common to both categories (Figure 4A). The limited overlap was corroborated by a principal component analysis (PCA) showing that $CSL^{-/-}$ cells clustered quite distinctly from control cells, and that ligand activation and DAPT treatment had quite limited effects on the transcriptomes in the $CSL^{-/-}$ cells, whereas the effect was more profound in the control cells (Figure 4B).

To assess how CSL deficiency affected the transition from in vitro culture to the in vivo tumor situation, we compared the transcriptome from in vitro culturing with that from tumors at early and late stages after xenografting, using the S^3 technology to bioinformatically sort out the tumor (human) from the stromal (mouse) transcripts (Chivukula et al., 2015). PCA revealed that the in vitro transcriptomes from MDA-MB-231$^{CSL-/-}$ and control cells were quite distinct and that the differences were maintained in the tumor situation (Figure 4C). Interestingly, when tumor cells were excised and returned to in vitro culture the transcriptomes largely reverted back to more closely resemble the respective in vitro transcriptomal profile observed prior to transplantation (Figure 4C). Finally, single-cell transcriptome analysis showed that MDA-MB-231$^{CSL-/-}$-derived tumors were more homogeneous than MDA-MB-231$^{CSL+/+}$-derived tumors, and cellular homogeneity further increased at the later tumor stage (Figure 4D). In sum, these data suggest that CSL transcriptionally controls a number of genes that are not part of a core Notch signature.

DISCUSSION

CSL serves as the central node in canonical Notch signaling by transmitting signaling from all Notch receptors upon ligand activation. In this work, we report that genetic ablation of CSL in breast tumor cells leads to enhanced tumor growth after transplantation into mammary fat pads in mice, an unexpected finding given that blocking of Notch at the receptor level reduces and activation of Notch promotes tumor growth (Bolós et al., 2013; Suman et al., 2013).

The genome-wide transcriptome data support the view that CSL does not merely mediate Notch signaling, as the set of genes upregulated by CSL ablation was considerably larger than the Notch signature in the MDA-MB-231 cells. This conclusion differs from a recent report,

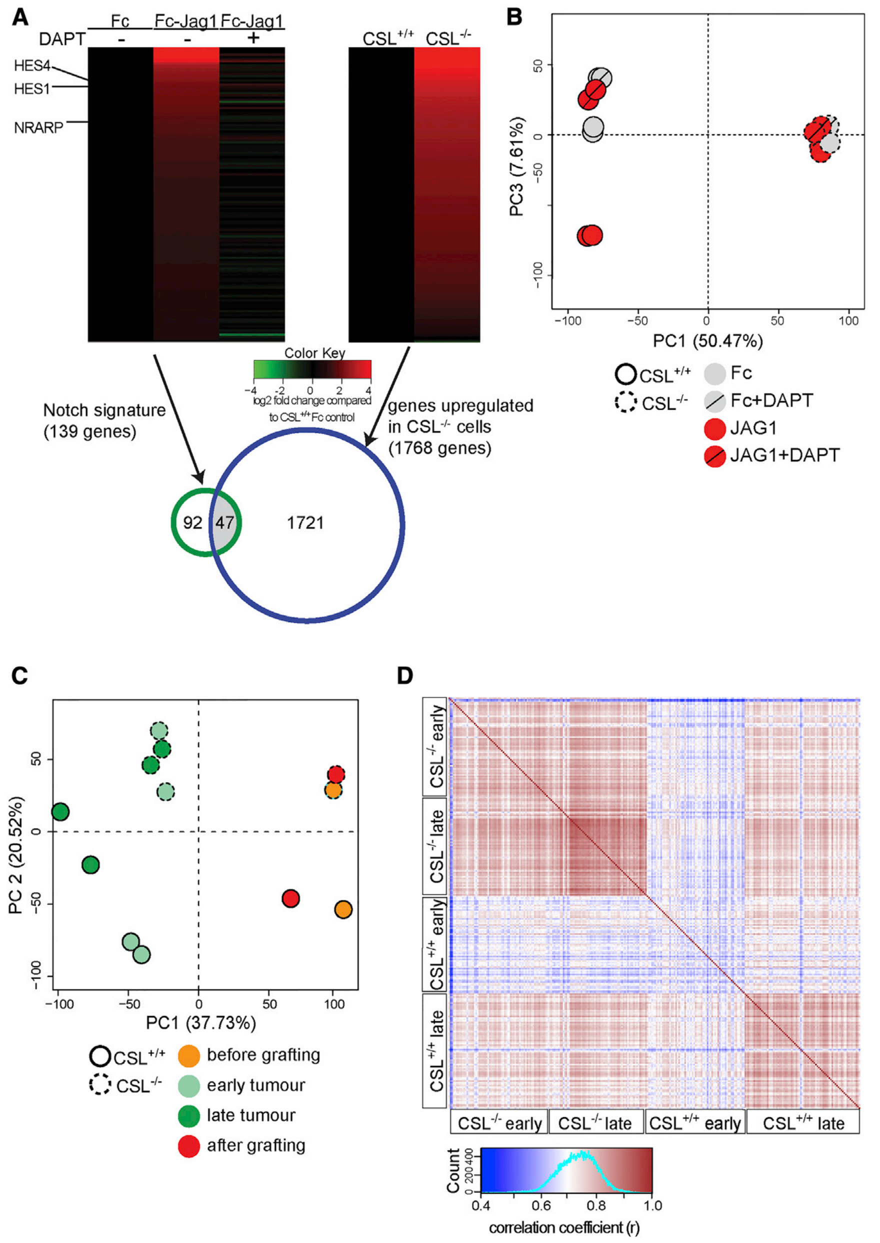

Figure 4. A Notch-Independent Transcriptional Signature in the CSL-Deficient Cells

(A) (Left) Heatmap of the 139 genes that constitute the Notch signature, i.e., genes which are upregulated by ligand (Jag1) stimulation and where gene expression is abrogated by DAPT. (Right) Heatmap of the 1,768 genes that were upregulated in CSL-deficient cells compared with control cells. Lower panel: Venn diagram showing the comparison of the Notch signature genes and genes upregulated in CSL-deficient cells.

(B) PCA of genome-wide transcriptome analysis for $CSL^{+/+}$ and $CSL^{-/-}$ cells during ligand activation (Jag1) and inhibition by DAPT (n = 2 for each treatment).

(C) PCA of genome-wide transcriptome analysis for $CSL^{+/+}$ and $CSL^{-/-}$ cells during in vitro culturing prior to tumor xenografting, the xenograft tumors, and when returned to in vitro culture after xenografting, as indicated.

(D) Correlation plot of single-cell RNA-seq analysis from $CSL^{+/+}$ and $CSL^{-/-}$ cells in tumors from early (3 weeks) and late (5 weeks) stages.

which used small hairpin RNA to knock down CSL expression in MDA-MB-231 cells (Kulic et al., 2014). While Kulic et al. (2014), like us, showed that reduced CSL expression in MDA-MB-231 cells promotes tumor growth, they argue that the observed phenotype was linked to derepression of Notch-activated genes. This notion, however, was based on gene-expression analysis of a set of only 170 genes that were on theoretical grounds considered to be Notch responsive (Kulic et al., 2014). Only five of the 170 genes, however, were among the 1,768 genes upregulated in the $CSL^{-/-}$ cells and only two genes were common to the 139 genes in our Notch signature (Figures S4B and S4C). The notion that CSL has Notch-independent functions is in line with the fact that CSL interacts with a number of proteins that are not linked to Notch signaling, such as CTCF, EBNA3c, interferon regulatory factor 4, and RITA (Collins et al., 2014). One hypothesis to explain the large Notch-independent gene set posits that CSL binds to a larger number of genomic sites, only a subset of which can bind Notch ICD, and loss of CSL would thus lead to derepression also of Notch-independent genes. Recent data, however, support a view whereby CSL is dynamically recruited by Notch ICD and not statically bound as a repressor (Castel

et al., 2013; Krejcí and Bray, 2007). An alternative hypothesis is that CSL may not directly repress all genes whose expression is altered, but rather control expression of a smaller set of chromatin modifiers or transcriptional regulators, which in turn reset the chromatin landscape and/or alter gene expression on a broader scale. In support of this view, we noted that expression of a number of such factors, such as Serpin, was upregulated in the CSL-deficient cells. That loss of CSL enhances tumor development is further underlined by a recent study reporting that combined silencing of CSL and p53 in cancer-associated fibroblasts leads to stromal and cancer cell expansion (Procopio et al., 2015).

An unexpected consequence of removing CSL was the unlocking of a hypoxic response during normoxia, manifested by a post-transcriptional elevation of HIF1α protein levels and the upregulation of hypoxia-regulated genes such as *VEGF-A*, *STC2*, and *KLF8*. This is in line with previous reports on normoxic HIF1α protein stabilization (Palmer et al., 2000; Ranasinghe et al., 2015; for review see Kuschel et al., 2012), and adds to the emerging view of a multifaceted interplay between Notch and the cellular hypoxic response (Andersson and Lendahl, 2014; Lendahl et al., 2009). Notch1 ICD interacted with HIF1α, and it is noteworthy that blocking Notch ICD generation reduced the amount of HIF1α, raising the intriguing possibility that Notch ICD in some way plays a role in the observed HIF1α stabilization. Furthermore, the HIF1α protein levels in the CSL-deficient cells were reduced by DTT treatment, suggesting a role for redox potential, possibly linked to nitrosylation of HIF1α (Palmer et al., 2000) or destabilization of the ODD domain. The unleashing of a hypoxic response during normoxia may also be linked to the acquisition of aberrant cell morphology, a phenotype strongly reminiscent of the recently described polyploid giant cancer cell (PGCC) phenotype (Zhang et al., 2014). Interestingly, PGCC cells, which are endowed with cancer stem cell-like properties, were recently identified in a number of tumor contexts in response to hypoxia or chemical induction of the hypoxic response by $CoCl_2$ (Zhang et al., 2014), suggesting that loss of CSL may lead to a PGCC-like state via upregulation of HIF1α. As PGCC cells are endowed with reduced proliferative rate in vitro combined with accelerated tumor growth capacity (Zhang et al., 2014), the induction of HIF1α protein levels in the CSL-deficient cells may underlie their enhanced growth rates, invasive capacity, and accelerated tumor growth.

In conclusion, the data in this report provide evidence for a role for CSL in controlling the cellular hypoxic response and cell cycle/cytokinesis as well as tumor growth. The data also indicate that CSL acts beyond only mediating Notch signaling.

EXPERIMENTAL PROCEDURES

CRISPR/Cas9 Genome Editing

Designed guide RNA sequence targeting CSL (5′-AAACATTGTATATATCTGAC-3′) was cloned and ligated to the guide RNA vector (Addgene). Cells were co-transfected with the guide RNA vector and the Cas9 expression vector. Single-cell colonies were isolated and subjected to Western blot and DNA-sequencing analysis.

Mammary Fat Pad Xenograft

All animal procedures were approved by the Stockholm's North Ethical Committee for Animal Research (permit No N151/14). 1.5×10^6 MDA-MB-231 control or CSL-deficient cells in culture media were orthotopically injected into the left and right fourth inguinal mammary fat pads of 4- to 6-week-old female immunodeficient NOD/SCID mice. Tumor growth and size was measured twice per week using calipers. Tumor volume was calculated according to the formula $L \times W^2$. Mice were euthanized at the third and fifth week after transplantation.

Statistical Analysis

All statistical analyses were calculated by GraphPad Prism (ver. 6). For further details see Supplemental Information.

ACCESSION NUMBERS

The accession number for the RNA-seq reported in this paper is GEO: GSE77308.

SUPPLEMENTAL INFORMATION

Supplemental Information includes Supplemental Experimental Procedures, four figures, three tables, and one prism file and can be found with this article online at http://dx.doi.org/10.1016/j.stemcr.2016.03.004.

AUTHOR CONTRIBUTIONS

All authors fulfill the ICMJE guidelines for authorship. E.B.B., Y.L.T., Y.P.P., S.L., H.S.C., Q.D., and S.-B.J. performed the biological experiments. Y.L.T. and D.R. performed the bioinformatics part of the study. A.L., X.L., C.S., S.-B.J., and U.L. designed the study. S.-B.J. and U.L. conceived the study and U.L. was the lead writer of the manuscript. All authors critically read the manuscript, approved the final version, and agree to be accountable for all aspects of the work. Shared first authors: E.B.B. (Figures 2 and S2), Y.L.T. (Figures 4 and S4), Y.P.P. (Figures 1D and 1E).

ACKNOWLEDGMENTS

We wish to thank Agneta Birgitta Andersson and the BRECT core facility for excellent assistance with the xenograft experiments. This work was financially supported by the Swedish Cancer Society, the Swedish Research Council (DBRM and Project Grant), Knut och Alice Wallenbergs Stiftelse, Karolinska Institutet (BRECT), the Swedish Cultural Foundation of Finland, and K. Albin Johansson Foundation.

Received: July 30, 2015
Revised: March 8, 2016
Accepted: March 8, 2016
Published: April 7, 2016

REFERENCES

Andersson, E.R., and Lendahl, U. (2014). Therapeutic modulation of Notch signalling—are we there yet? Nat. Rev. Drug Discov. *13*, 357–378.

Andersson, E.R., Sandberg, R., and Lendahl, U. (2011). Notch signaling: simplicity in design, versatility in function. Development *138*, 3593–3612.

Bolós, V., Mira, E., Martínez-Poveda, B., Luxán, G., Cañamero, M., Martínez-A, C., Mañes, S., and de la Pompa, J.L. (2013). Notch activation stimulates migration of breast cancer cells and promotes tumor growth. Breast Cancer Res. *15*, R54.

Borggrefe, T., and Oswald, F. (2014). Keeping notch target genes off: a CSL corepressor caught in the act. Structure *22*, 3–5.

Castel, D., Mourikis, P., Bartels, S.J.J., Brinkman, A.B., Tajbakhsh, S., and Stunnenberg, H.G. (2013). Dynamic binding of RBPJ is determined by Notch signaling status. Genes Dev. *27*, 1059–1071.

Chivukula, I.V., Ramsköld, D., Storvall, H., Anderberg, C., Jin, S., Mamaeva, V., Sahlgren, C., Pietras, K., Sandberg, R., and Lendahl, U. (2015). Decoding breast cancer tissue-stroma interactions using species-specific sequencing. Breast Cancer Res. *17*, 109.

Collins, K.J., Yuan, Z., and Kovall, R. (2014). Structure and function of the CSL-KyoT2 corepressor complex: a negative regulator of Notch signaling. Structure *22*, 70–81.

Conlon, R., Reaume, G., and Rossant, J. (1995). Notch1 is required for the coordinate segmentation of somites. Development *121*, 1533–1545.

Guruharsha, K.G., Kankel, M.W., and Artavanis-Tsakonas, S. (2012). The Notch signalling system: recent insights into the complexity of a conserved pathway. Nat. Rev. Genet. *13*, 654–666.

Gustafsson, M.V., Zheng, X., Pereira, T., Gradin, K., Jin, S., Lundkvist, J., Ruas, J.L., Poellinger, L., Lendahl, U., and Bondesson, M. (2005). Hypoxia requires notch signaling to maintain the undifferentiated cell state. Dev. Cell *9*, 617–628.

Hansson, E.M., Teixeira, A.I., Gustafsson, M.V., Dohda, T., Chapman, G., Meletis, K., Muhr, J., and Lendahl, U. (2006). Recording Notch signaling in real time. Dev. Neurosci. *28*, 118–127.

Holliday, D.L., and Speirs, V. (2011). Choosing the right cell line for breast cancer research. Breast Cancer Res. *13*, 215.

Jain, R.K. (2014). Antiangiogenesis strategies revisited: from starving tumors to alleviating hypoxia. Cancer Cell *26*, 605–622.

Jin, S., Mutvei, P., Chivukula, I.V., Andersson, E.R., Ramsköld, D., Sandberg, R., Lee, K.L., Kronqvist, P., Mamaeva, V., Ostling, P., et al. (2013). Non-canonical Notch signaling activates IL-6/JAK/STAT signaling in breast tumor cells and is controlled by p53 and IKKα/IKKβ. Oncogene *32*, 4892–4902.

Krejcí, A., and Bray, S. (2007). Notch activation stimulates transient and selective binding of Su(H)/CSL to target enhancers. Genes Dev. *21*, 1322–1327.

Kulic, I., Robertson, G., Chang, L., Baker, J.H.E., Lockwood, W.W., Mok, W., Fuller, M., Fournier, M., Wong, N., Chou, V., et al. (2014). Loss of the Notch effector RBPJ promotes tumorigenesis. J. Exp. Med. *212*, 37–52.

Kuschel, A., Simon, P., and Tug, S. (2012). Functional regulation of HIF-1α under normoxia—is there more than post-translational regulation? J. Cell. Physiol. *227*, 514–524.

Lake, R.J., Tsai, P.F., Choi, I., Won, K.J., and Fan, H.Y. (2014). RBPJ, the major transcriptional effector of notch signaling, remains associated with chromatin throughout mitosis, suggesting a role in mitotic bookmarking. PLoS Genet. *10*, e1004204.

Lendahl, U., Lee, K.L., Yang, H., and Poellinger, L. (2009). Generating specificity and diversity in the transcriptional response to hypoxia. Nat. Rev. Genet. *10*, 821–832.

Lowell, S., Benchoua, A., Heavey, B., and Smith, A.G. (2006). Notch promotes neural lineage entry by pluripotent embryonic stem cells. PLoS Biol. *4*, e121.

Maekawa, Y., Ishifune, C., Tsukumo, S., Hozumi, K., Yagita, H., and Yasutomo, K. (2015). Notch controls the survival of memory CD4+ T cells by regulating glucose uptake. Nat. Med. *21*, 1–9.

Mutvei, A.P., Fredlund, E., and Lendahl, U. (2015). Frequency and distribution of Notch mutations in tumor cell lines. BMC Cancer *15*, 311.

Nam, Y., Sliz, P., Song, L., Aster, J.C., and Blacklow, S.C. (2006). Structural basis for cooperativity in recruitment of MAML coactivators to Notch transcription complexes. Cell *124*, 973–983.

Oka, C., Nakano, T., Wakeham, A., de la Pompa, J.L., Mori, C., Sakai, T., Okazaki, S., Kawaichi, M., Shiota, K., Mak, T.W., et al. (1995). Disruption of the mouse RBP-J kappa gene results in early embryonic death. Development *121*, 3291–3301.

Palmer, L., Gaston, B., and Johns, R. (2000). Normoxic stabilization of hypoxia-inducible factor-1 expression and activity: redox-dependent effect of nitrogen oxides. Mol. Pharmacol. *58*, 1197–1203.

Procopio, M.-G., Laszlo, C., Al Labban, D., Kim, D.E., Bordignon, P., Jo, S.-H., Goruppi, S., Menietti, E., Ostano, P., Ala, U., et al. (2015). Combined CSL and p53 downregulation promotes cancer-associated fibroblast activation. Nat. Cell. Biol. *17*, 1193–1204.

Ranasinghe, W.K.B., Baldwin, G.S., Bolton, D., Shulkes, A., Ischia, J., and Patel, O. (2015). HIF1α expression under normoxia in prostate cancer—which pathways to target? J. Urol. *193*, 763–770.

Rapisarda, A., and Melillo, G. (2012). Overcoming disappointing results with antiangiogenic therapy by targeting hypoxia. Nat. Rev. Clin. Oncol. *9*, 378–390.

Robinson, D.R., Kalyana-Sundaram, S., Wu, Y.-M., Shankar, S., Cao, X., Ateeq, B., Asangani, I., Iyer, M., Maher, C., Grasso, C.S., et al. (2011). Functionally recurrent rearrangements of the MAST kinase and Notch gene families in breast cancer. Nat. Med. *17*, 1646–1651.

Sahlgren, C., Gustafsson, M.V., Jin, S., Poellinger, L., and Lendahl, U. (2008). Notch signaling mediates hypoxia-induced tumor cell migration and invasion. Proc. Natl. Acad. Sci. USA *105*, 6392–6397.

Schmitt, T.M., de Pooter, R.F., Gronski, M., Cho, S.K., Ohashi, P.S., and Zúñiga-Pflücker, J.C. (2004). Induction of T cell development

and establishment of T cell competence from embryonic stem cells differentiated in vitro. Nat. Immunol. *5*, 410–417.

Suman, S., Das, T.P., and Damodaran, C. (2013). Silencing NOTCH signaling causes growth arrest in both breast cancer stem cells and breast cancer cells. Br. J. Cancer *109*, 2587–2596.

Weng, A.P., Ferrando, A., Lee, W., Morris, J.P., Silverman, L.B., Sanchez-Irizarry, C., Blacklow, S.C., Look, T., and Aster, J.C. (2004). Activating mutations of NOTCH1 in human T cell acute lymphoblastic leukemia. Science *306*, 269–271.

Westhoff, B., Colaluca, I.N., D'Ario, G., Donzelli, M., Tosoni, D., Volorio, S., Pelosi, G., Spaggiari, L., Mazzarol, G., Viale, G., et al. (2009). Alterations of the Notch pathway in lung cancer. Proc. Natl. Acad. Sci. USA *106*, 22293–22298.

Wilson, J.J., and Kovall, R. (2006). Crystal structure of the CSL-Notch-Mastermind Ternary complex bound to DNA. Cell *124*, 985–996.

Yin, X., Farin, H.F., van Es, J.H., Clevers, H., Langer, R., and Karp, J.M. (2014). Niche-independent high-purity cultures of Lgr5+ intestinal stem cells and their progeny. Nat. Methods *11*, 106–112.

Zhang, S., Mercado-Uribe, I., Xing, Z., Sun, B., Kuang, J., and Liu, J. (2014). Generation of cancer stem-like cells through the formation of polyploid giant cancer cells. Oncogene *33*, 116–128.

Zheng, X., Linke, S., Dias, J.M., Zheng, X., Gradin, K., Wallis, T.P., Hamilton, B.R., Gustafsson, M., Ruas, J.L., Wilkins, S., et al. (2008). Interaction with factor inhibiting HIF-1 defines an additional mode of cross-coupling between the Notch and hypoxia signaling pathways. Proc. Natl. Acad. Sci. USA *105*, 3368–3373.

Stem Cell Reports, Volume *6*

Supplemental Information

Loss of CSL Unlocks a Hypoxic Response and Enhanced Tumor Growth Potential in Breast Cancer Cells

Eike-Benjamin Braune, Yat Long Tsoi, Yee Peng Phoon, Sebastian Landor, Helena Silva Cascales, Daniel Ramsköld, Qiaolin Deng, Arne Lindqvist, Xiaojun Lian, Cecilia Sahlgren, Shao-Bo Jin, and Urban Lendahl

Figure S1

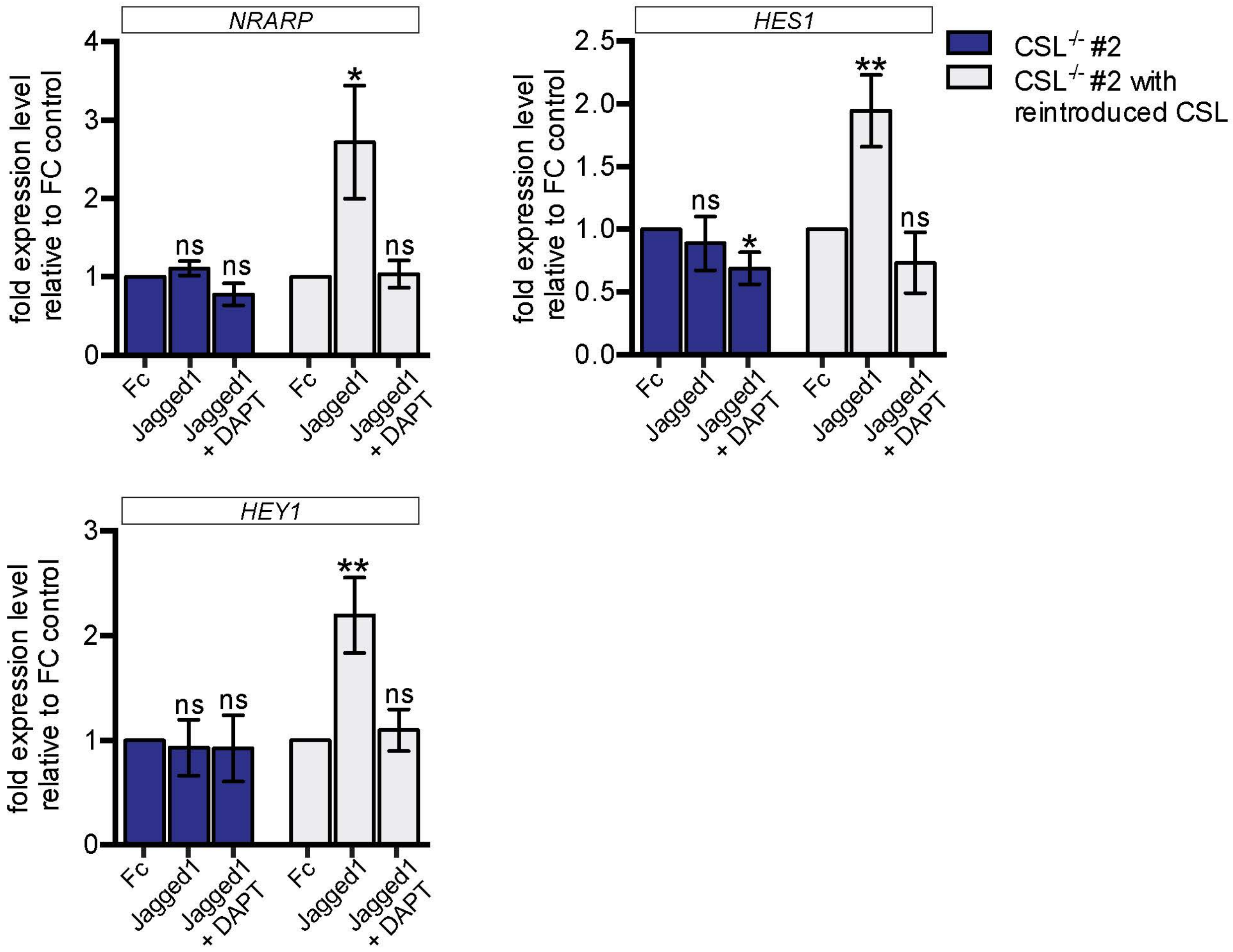

NRARP
HES1
HEY1
fold expression level relative to FC control
CSL-/- #2
CSL-/- #2 with reintroduced CSL
Fc
Jagged1
Jagged1 + DAPT
ns
*
**

Figure S2

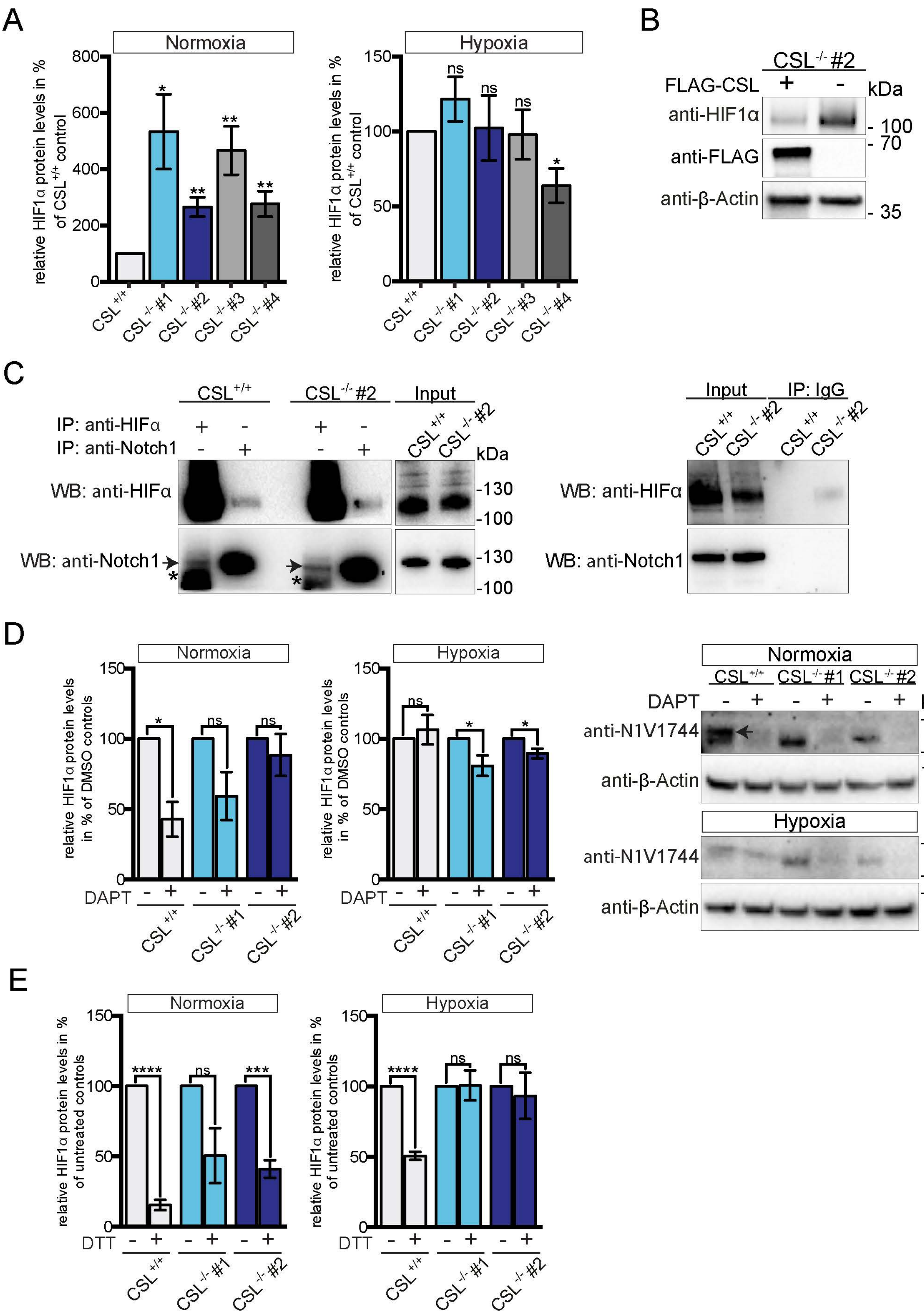
A
Normoxia
Hypoxia
relative HIF1α protein levels in % of CSL+/+ control
CSL+/+
CSL-/- #1
CSL-/- #2
CSL-/- #3
CSL-/- #4
B
CSL-/- #2
FLAG-CSL
anti-HIF1α
anti-FLAG
anti-β-Actin
kDa
C
CSL+/+
CSL-/- #2
Input
IP: anti-HIFα
IP: anti-Notch1
WB: anti-HIFα
WB: anti-Notch1
IP: IgG
D
Normoxia
Hypoxia
relative HIF1α protein levels in % of DMSO controls
DAPT
anti-N1V1744
anti-β-Actin
E
relative HIF1α protein levels in % of untreated controls
DTT

Figure S3

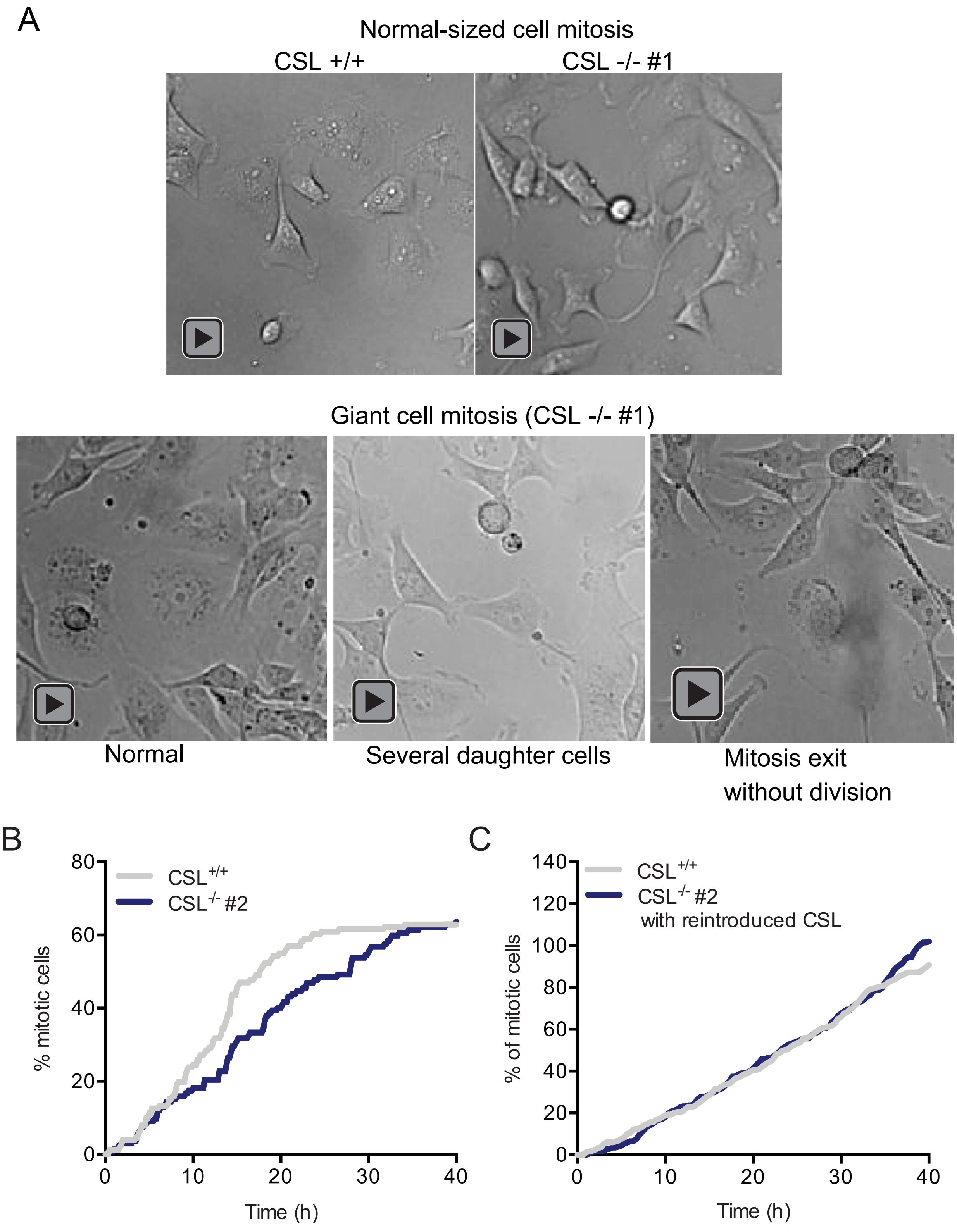
A
Normal-sized cell mitosis
CSL +/+
CSL -/- #1
Giant cell mitosis (CSL -/- #1)
Normal
Several daughter cells
Mitosis exit without division
B
CSL+/+
CSL-/- #2
% mitotic cells
Time (h)
0
10
20
30
40
60
80
C
CSL+/+
CSL-/- #2
with reintroduced CSL
% of mitotic cells
Time (h)
100
120
140

Figure S4

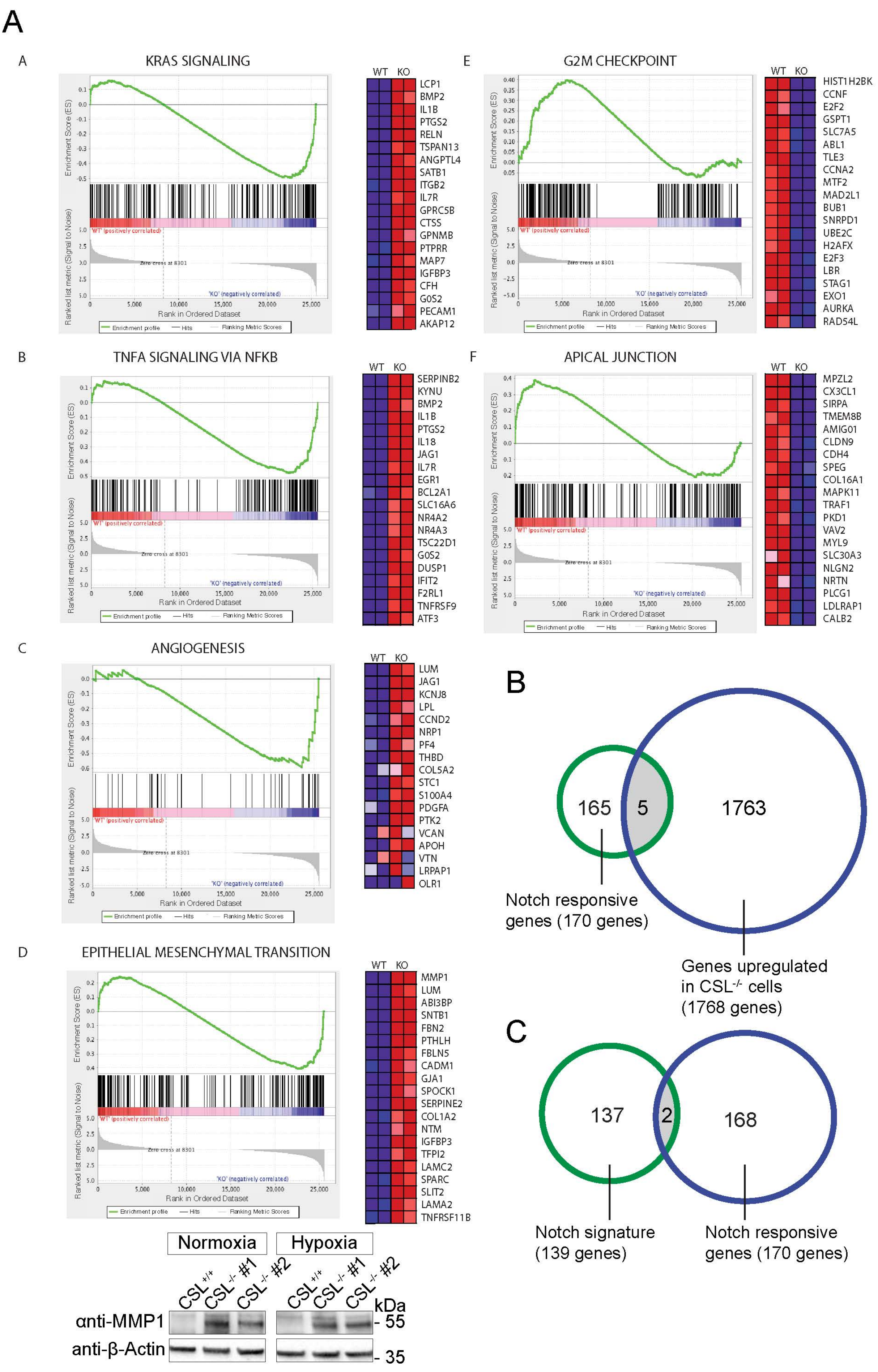
A
A
KRAS SIGNALING
WT KO
LCP1
BMP2
IL1B
PTGS2
RELN
TSPAN13
ANGPTL4
SATB1
ITGB2
IL7R
GPRC5B
CTSS
GPNMB
PTPRR
MAP7
IGFBP3
CFH
G0S2
PECAM1
AKAP12
E
G2M CHECKPOINT
WT KO
HIST1H2BK
CCNF
E2F2
GSPT1
SLC7A5
ABL1
TLE3
CCNA2
MTF2
MAD2L1
BUB1
SNRPD1
UBE2C
H2AFX
E2F3
LBR
STAG1
EXO1
AURKA
RAD54L
B
TNFA SIGNALING VIA NFKB
WT KO
SERPINB2
KYNU
BMP2
IL1B
PTGS2
IL18
JAG1
IL7R
EGR1
BCL2A1
SLC16A6
NR4A2
NR4A3
TSC22D1
G0S2
DUSP1
IFIT2
F2RL1
TNFRSF9
ATF3
F
APICAL JUNCTION
WT KO
MPZL2
CX3CL1
SIRPA
TMEM8B
AMIGO1
CLDN9
CDH4
SPEG
COL16A1
MAPK11
TRAF1
PKD1
VAV2
MYL9
SLC30A3
NLGN2
NRTN
PLCG1
LDLRAP1
CALB2
C
ANGIOGENESIS
WT KO
LUM
JAG1
KCNJ8
LPL
CCND2
NRP1
PF4
THBD
COL5A2
STC1
S100A4
PDGFA
PTK2
VCAN
APOH
VTN
LRPAP1
OLR1
D
EPITHELIAL MESENCHYMAL TRANSITION
WT KO
MMP1
LUM
ABI3BP
SNTB1
FBN2
PTHLH
FBLN5
CADM1
GJA1
SPOCK1
SERPINE2
COL1A2
NTM
IGFBP3
TFPI2
LAMC2
SPARC
SLIT2
LAMA2
TNFRSF11B
Enrichment Score (ES)
Ranked list metric (Signal to Noise)
'WT' (positively correlated)
Zero cross at 8301
'KO' (negatively correlated)
Rank in Ordered Dataset
Enrichment profile
Hits
Ranking Metric Scores
B
165
5
1763
Notch responsive genes (170 genes)
Genes upregulated in CSL-/- cells (1768 genes)
C
137
2
168
Notch signature (139 genes)
Notch responsive genes (170 genes)
Normoxia
Hypoxia
CSL+/+
CSL-/- #1
CSL-/- #2
kDa
anti-MMP1
anti-β-Actin
- 55
- 35

Supplemental Figure legends

Figure S1. Quantitative PCR analysis of *NRARP, HES1* and *HEY1* mRNA expression in $CSL^{-/-}$ #2 cells and in $CSL^{-/-}$ #2 expressing reintroduced FLAG-CSL after activation of Notch (Jagged1) or inhibition of Notch receptor cleavage (Jagged1 + DAPT). mRNA expression level analysis is based on three separate experiments. Data are presented as mean ± SD. * $P \leq 0.05$; ** $P \leq 0.01$.

Figure S2. (A) Quantification of Western blots presented in Figure 2A. **(B)** Western blot of HIFα, FLAG and β-actin (loading control) of lysates from CSL-deficient cells (clone #2) and cells expressing reintroduced FLAG-CSL. **(C)** Immunoprecipitation of endogenous Notch1 or HIFα from control or CSL-deficient cells. Immunoblotting was performed with Notch1 and Hifa antibodies, as indicated. The arrow denotes the Notch1 ICD band and the asterisk (*) denotes a band of unknown nature. **(D)** (left) Quantification of Western blots presented in Figure 2E. (right) Western blot of cleaved Notch1 and β-actin (loading control) in control and CSL-deficient MDA-MB-231 cells under normoxic (top) and hypoxic conditions (bottom) with and without DAPT treatment. The arrow denotes a band of unknown nature that is uniquely present in $CSL^{+/+}$ cells. **(E)** Quantification of Western blots shown in Figure 2F. All graphs presenting quantification of protein levels are based on at least three independent experiments. Data are presented as mean ± SEM. * $P \leq 0.05$; ** $P \leq 0.01$; *** $P \leq 0.001$; **** $P \leq 0.0001$.

Figure S3. (A) Live cell imaging experiments showing cell division and proliferation of control and CSL-deficient cells. **(B)** Graph showing the cumulative mitotic entry of at least 100 cells per condition. The cell cycle time for $CSL^{+/+}$ cells is approximately 30h, the cell cycle time for $CSL^{-/-}$ #2 cells is approximately 50-55h. **(C)** Graph showing the cumulative mitotic entry of control and $CSL^{-/-}$ #2 cells expressing reintroduced FLAG-CSL.

Figure S4. (A) GSEA analysis of $CSL^{+/+}$ and $CSL^{-/-}$ cells. (bottom) Western blot of MMP1 and β-actin (loading control) protein levels in control and CSL-deficient MDA-MB-231 cells under normoxic and hypoxic conditions. Note that MMP1 is only barely detectable in control cells.Venn diagrams: **(B)** the 1768 genes upregulated in the CSL-deficient cells compared to the set of 170 Notch-responsive genes defined by Kulic et al., (2014); **(C)** the 139 genes defined as the Notch signature in this study to the 170 genes defined by Kulic et al., (2014).

Supplemental Experimental Procedures

Cell culturing and transfections

MDA-MB-231 were purchased from American Type Culture Collection (ATCC) and maintained in Dulbecco's Modified Eagle Medium (DMEM) with 10% fetal bovine serum (FBS) and 1% penicillin-streptomycin (Life Techonologies). Cells were transfected using lipofectamine 2000 (Life Technologies) according to the manufacturer's protocols. To block Notch signaling cells were treated with 10 µM DAPT overnight. To activate Notch signaling cells were mechanical dissociated and seeded on 12 well plate precoated with control Fc or Jagged1-Fc (Jag) fragment for 7h.

Genomic DNA purification and sequencing

Genomic DNA from MDA-MB-231 CSL-deficient cells was isolated and purified using the GeneJET Genomic DNA Purification Kit (Life technologies). The CSL sequence targeted by the CRISPR constructs was amplified by PCR, using 40 ng of genomic DNA and a KOD DNA Polymerase (Merck Millipore). PCR was performed for 35 cycles, and the purified PCR product was sequenced. PCR primers used are listed in the respective Supplemental Table.

qRT-PCR

For qRT-PCR, 500ng of RNA was reverse-transcribed using *Maxima First Strand cDNA synthesis* kit (Thermo Scientific). qRT-PCR was performed on *Applied Biosystems 7500 Real-Time PCR System* (ABI 7500; Applied Biosystems) and gene expression was detected with Power SYBR Green (Applied Biosystems). Relative gene expression was determined by normalizing to GAPDH mRNA expression. Primers used in qRT-PCR are listed in the respective Supplemental Table.

Western blot

MDA-MB-231 control and CSL-deficient cells were cultured on a 100 mm culture dishes to 90% confluence and subjected to hypoxic conditions (1% oxygen) for 4-5h or left untreated in normoxic conditions. The cells were washed in ice-cold PBS and subsequently lysed in sample buffer [62.5 mM Tris-HCl, pH 6.8, 25% glycerol, 2% SDS, 5% β-mercaptoethanol, 0.01% bromphenolblue], heated for 10 min to 95°C and either loaded on Mini-Protean Tris-Glycine TGX gradient gels (Biorad) or stored at -80°C. Protein lysates were separated on Mini-Protean Tris-Glycine TGX gradient gels (Bio-rad). After electrophoresis, gels were placed directly onto a Trans-Blot Turbo PVDF membrane transfer pack and transferred using the "HIGH MW" protocol (Bio-Rad). Subsequently, membranes were blocked for 1h at RT using StartingBlock (PBS) buffer (Thermo Scientific) supplemented with 0.1% Tween20 (Sigma- Aldrich). Detection of antibody binding was performed by using Clarity Western ECL-Substrate (Bio-rad). All antibodies used are listed in the respective Supplemental Table. Proteins expression levels were quantified by using ImageJ64 (Ver. 1.48) with beta-Actin for normalization. For signal quantification only non-saturated bands were measured. All calculations are based on a minimum of three independent western blots.

Antibodies, immunohistochemistry and immunocytochemistry

Tumor samples were fixed with 4% paraformaldehyde at 4°C overnight, cytoprotected with 30% sucrose at 4°C overnight, embedded in OCT compound (Tissue-Tek) and sectioned at 8 µm. Sections were blocked in blocking solution (10% normal donkey serum, 1% bovine serum albumin (BSA) and 1% Triton-X (Sigma-Aldrich) in PBS) at room temperature for 1h and incubated with primary antibody diluted in blocking solution at 4°C overnight. Slides were washed with PBS three times five min each and incubated with fluorescence secondary antibody (Alexa Fluorophore) in blocking solution at room temperature for 1h. Slides were washed with PBS for five min three times and mounted with *Vectashield Antifade Mounting Medium with DAPI* (Vector Labs). Confocal images were taken with a Zeiss LSM 700 (Carl Zeiss). All antibodies used are listed in the respective Supplemental Table.

Hematoxylin and eosin (H&E) staining

Cryosection slides of 8 µm thickness were rehydrated in PBS for five min, stained with Gill's hematoxylin (Sigma-Aldrich) diluted 1:1 in PBS for five min and washed with running water for five min. Slides were then

stained with Eosin (Sigma-Aldrich) for 1.5 min, wash briefly in water, dehydrated in a series of dilution (70%, 95%, 95%, 100%, 100% ethanol, xylene, xylene) and mounted with *Organo/Limonene Mount* (Sigma-Aldrich).

Cellular hypoxia and γ-secretase inhibition

MDA-MB-231 control and CSL-deficient cells were cultured on 100 mm cell culture dishes to ca 70% confluence and subjected to hypoxic conditions (1% oxygen) for 48h or left untreated in normoxic conditions. During the incubation the cells were treated with 10 μM DAPT or DMSO as control. After 48h cells were harvested and whole cell lysates were prepared as described above.

Cellular hypoxia and DTT treatment

MDA-MB-231 control and CSL-deficient cells were cultured on 100 mm cell culture dishes to 95% confluence and subjected to hypoxic conditions (1% oxygen) for 4h or left untreated in normoxic conditions. During the incubation the cells were treated with 100 μM DTT or left untreated. After 4h cells were harvested and whole cell lysates were prepared as described above.

HIF1a-Notch1 Immunoprecipitation

MDA-MB-231 control and CSL-deficient cells were cultured on 100 mm cell culture dishes to 90% confluence and subjected to hypoxic conditions (1% oxygen) for 4h or left untreated in normoxic conditions. The cells were washed twice with ice-cold PBS, fixed with 1% PFA for 10 min at room temperature and subsequently incubated with 100 mM Glycine added directly to the medium for five min at room temperature to quench the reaction. The cells were then lysed in ice-cold lysis buffer [20 mM Tris-HCL pH 8, 125 mM NaCl, 2 mM EDTA, 0.025% deoxycholate (DOC), 0.025% SDS, 0.5% Nonidet-P40] supplemented with protease-inhibitor cocktail (CST) for 30 min at 4°C and sonicated using a *Bioruptor UCD-200* sonication device (Diagenode). In addition lysates were passed through a 23G needle attached to a 1 ml syringe to complete cell lysis. Lysates were clarified by 10 min of centrifugation at 10000 x *g* at 4°C. The supernatant was subsequently incubated with the indicated antibodies (see also Supplemental Table) overnight on an end-to-end rotator at 4°C. Normal rabbit IgG #2729 (CST) was used as control. 30 μl Sepharose-G-beads (GE Healthcare) were added to each immunoprecipitation and incubated for 4h at 4°C on an end-to-end rotator. The beads were washed once in low-salt immune complex wash buffer (upstate) and two times in lysis buffer, 20 min each. Dry beads were mixed with sample buffer [62.5 mM Tris-HCl, pH 6.8, 25% glycerol, 2% SDS, 5% β-mercaptoethanol, 0.01% bromphenolblue], heated for 10 min to 95°C and either loaded on Mini-Protean Tris-Glycine TGX gradient gels (Biorad) or stored at -80°C.

Cell migration and invasion assays

Cell migration was determined *in vitro* by using 8.0 mm pore polycarbonate membrane *Corning transwell* inserts (BD Biosciences) according to the manufacturers' instruction. Cells were starved overnight in serum-free media. Then, 2 x 10^4 cells were seeded into the upper transwell chamber with serum-free media containing DMSO or 10 μM DAPT, while media with 10% FBS was added into the lower chamber as chemoattractant and cells were allowed to migrate over night. Cell invasion was determined *in vitro* by utilizing 8.0 mm pore *BD BioCoat Matrigel Invasion Chambers* (BD Biosciences) according to the manufacturers' instruction: 2 x 10^4 cells were seeded into the upper chamber and allowed to invade through the Matrigel-coated membrane under the influence of 10% FBS containing media at the lower chamber for over night. Cells that had migrated across the membrane in both assays were fixed with 4% paraformaldehyde and stained with DAPI. Excess cells were removed using a cotton swab. For each treatment six randomly chosen images were taken. All cells were imaged using a Zeiss Axiovert A1 (Carl Zeiss). Images were analyzed and counted by CellProfiler Ver. 2.1.1 image analysis software (Broad Institute Inc.) with default settings.

In ovo tumor growth

Fertilized chicken eggs were placed in an egg incubator under rotation at 37°C with 60% humidity on day one of embryonic development. On day three eggs were turned, taken off rotation and punctured with a small hole, then covered with adhesive tape. On day eight the holes were expanded and a small plastic ring (5-6 mm in diameter) was placed on top of the chorioallantoic membrane (CAM). 1-2x10^6 MDA-MB-231 control or CSL-deficient cells were suspended in 1:1 PBS and matrigel for a total volume of 30 μl/egg. The cell suspensions were transplanted inside the plastic ring on the CAM and the eggs were covered with parafilm. On day 13 the tumors

were excised and fixed in 3% PFA for four hours at room temperature after which the tumors were dehydrated with ethanol series of 50%, 70%, 70% for one hour each. The tumors were visualized on a Zeiss SteREO Lumar V12 microscope with 0.8x NeoLumar objective and weighed using an analytical laboratory scale.

Transcriptome analysis and single cell RNA-seq

1. RNA preparation. Tumors with surrounding stromal tissues from xenografts were minced and non-enzymatically digested using cell dissociation enzyme-free PBS-based (Gibco) for 30 minutes at 37°C with occasional pipetting. The dissociated cells were then filtered through a 40 mm nylon mesh strainer. Single cell suspension was obtained by re-suspension in CO_2-independent medium (Gibco) for single cell picking. Single cells of xenografts were manually picked under the microscope as previously described (Picelli et al., 2013). Briefly, a single cell was picked using mouth pipette under 20X magnification in 0.5 ml solution. The picked single cell was released into RNase-free PCR strip tube containing hypotonic Smart-seq RNA lysis buffer consisting of 2.3 ml of 19:1 ratio of 0.4% TritonX-100 and RNase inhibitor (Clontech), 1 ml of 10 mM dNTP (Fermentas), 1 ml of 10 mM oligo-dT primer, and ERCC spike-in (Ambion) at 72°C for three min.

2. RNA-seq library preparation. Total RNA from tumors and cells was purified using the *RNeasy Mini* Kit (QIAGEN), according to the manufacturer's instructions. RNA concentration and RNA integrity number (RIN) were calculated by using the *Agilent RNA 6000 Nano* Kit (Agilent Technologies). Two biological replicates for each RNA sample were processed to cDNA library preparations using the *Illumina TruSeq*™ *RNA sample preparation* kit (Low-Throughput protocol) according to the manufacturer's protocol. Quantification and quality control of the cDNA libraries were done using the *Agilent DNA 1000* Kit (Agilent Technologies). The libraries were sequenced on an *Illumina HiSeq 2000* system.

3. Reverse transcription and PCR Pre-amplification. Reverse transcription of single cell RNA and PCR pre-amplification were performed according to previously described procedure with the specified conditions (Picelli et al., 2013). Briefly, single cell lysates were denatured at 72°C for three min, and followed by first-strand conversion in a reaction mixture of SuperScript II reverse transcriptase (Invitrogen), RNase inhibitor (Clontech), 5X SuperScript II First-Strand buffer (Invitrogen), DTT (Invitrogen), 5M Betaine (Sigma), MgCl2 (Sigma), and custom locked nucleic acid oligonucleotides. Reverse transcription was performed at 42°C for 90 min, followed by 10 cycles of (50°C for two min, 42°C for two min), and inactivation at 70°C for 15 min.

PCR reactions were performed directly after reverse transcription as described previously with the specified condition (Picelli et al., 2013). Briefly, the total volume of cDNA was added into PCR master mix containing KAPA HiFi HotStart ReadyMix (KAPA Biosystems) and ISPCR primers. The PCR reaction was performed at 98°C three min, then 18 cycles of (98°C for 15s, 67°C for 20s, 72°C for six min), and a final extension at 72°C for five min. Next, PCR products were purified using a 1:1 ratio of AMPure XP beads (Beckman Coulter), with the final elution of 15 ml of EB solution (Qiagen). The library quality and size distribution was checked using a High-Sensitivity DNA chip (Agilent Bioanalyzer).

4. Tagmentation and Final PCR amplification. One nanogram of cDNA was used for tagmentation reaction at 55°C for five min in a 20 ml 5X TAPS buffer, 5 ml PEG solution and 0.5 ml Tn5 transposase (Illumina). Then, 5 ml of 0.5% SDS was directly added into the tagmentation reaction and incubated at room temperature for five min. Final PCR amplification was carried out in a 25 ml of 10mM dNTP, 10 ml of 5X PCR buffer, 1 ml of KAPA polymerase (KAPA Biosystems) and 5 ml Index 1 primers (i7) and 5 ml of Index 2 primers (i5) (Illumina). The reaction was performed at 72°C for three min, 95°C for 30s, and 10 cycles of (95°C for 10s, 55°C for 30s, 72°C for 30s), followed by 72°C for five min and 10°C for infinite. Purification was performed with a 1:1 ratio of AMPure XP beads with an elution of 15 ml and purified samples were quality checked on a High-Sensitivity DNA chip. Next, quantification was carried out using Qubit High-Sensitivity DNA kit (Invitrogen). Finally, libraries were diluted to a final concentration of 2 nM and pooled for sequencing using Illumina HiSeq 2000.

5. Bioinformatics Analysis. Bioinformatics Analysis. Differential gene expression analysis was performed with edgeR package (Robinson et al., 2010). Differentially expressed genes with statistical significance were identified with fold change higher than 1.5 and false discovery rate lower than 0.05. PCA were generated with RPKM of individual samples with FactoMineR package available in Bioconductor. Reads per kilobase per million (rpkm) were calculated for GSEA analysis (Mootha et al., 2003; Subramanian et al., 2005) with the default setting and a false discovery rate at 0.25. For other analysis, tag per millions (TPM) values were defined as (rpkm / total rpkm of a sample * 1000000). To analyze the heterogeneity of the single cell transcriptomic data, 5102 genes with the highest average TPM among all samples or in a single tumor were selected for Pearson's correlation and other analysis. The bioinformatics separation of mouse and human transcriptomes from tumor tissue was carried out as described in Chivukula et al., 2015. All RNA-seq data are available at the Gene Expression Omnibus (GEO) website (Edgar et al., 2002). The assigned accession number is GSE77308.

Live-cell microscopy
For live-cell imaging experiments, 4.000 MDA-MB-231$^{CSL-/-}$ and control cells were seeded in 96-well imaging plates (BD Falcon) 16h before imaging on a Leica DMI6000 Imaging System using a 20x objective. After microscopy, images were processed and analysed using ImageJ.

Statistical analysis
For proliferation and apoptosis analysis (Figure 1F and G, respectively) signals of at least four randomly chosen images from one tumor sample of each kind were counted and compared by unpaired t-test. For chorioallantoic tumor size (Figure 1H) analysis of at least five different tumors of each kind were measured and compared by unpaired t-test. Comparison of invasion and migration (Figure 1I,J) is based on at least three independent experiments and was analyzed by unpaired t-test. HIF1a protein levels (Figure 2A,E and F, Figure S2A,D and E) were compared using unpaired t-test and are based on at least three independent experiments. mRNA expression levels were analyzed using unpaired t-test and are based on three separate experiments (Figure 2B,C). Comparison of vascularisation (Figure 2D) is based on signal quantification of at least three randomly chosen images from one tumor sample of each kind and was analyzed by unpaired t-test.

Supplemental References:

Edgar R., Domrachev M., Lash AE. (2002) Gene Expression Omnibus: NCBI gene expression and hybridization array data repository. Nucleic Acids Res. 30, 207-10

Mootha, V. K., Lindgren, C. M., Eriksson, K.-F., Subramanian, A., Sihag, S., Lehar, J., et al. (2003). PGC-1alpha-responsive genes involved in oxidative phosphorylation are coordinately downregulated in human diabetes. Nat. Genet. *34*, 267–273.

Picelli, S., Björklund, Å. K., Faridani, O. R., Sagasser, S., Winberg, G., & Sandberg, R. (2013). Smart-seq2 for sensitive full-length transcriptome profiling in single cells. Nat. Methods. *10*, 1096–8.

Robinson, M. D., McCarthy, D. J., & Smyth, G. K. (2010). edgeR: a Bioconductor package for differential expression analysis of digital gene expression data. Bioinformatics *26*, 139–140.

Subramanian, A., Tamayo, P., Mootha, V. K., Mukherjee, S., Ebert, B. L., Gillette, M., et al. (2005). Gene set enrichment analysis: a knowledge-based approach for interpreting genome-wide expression profiles. Proc. Natl. Acad. Sci. U. S. A. *102*, 15545–50.
